# *Myt1l* haploinsufficiency leads to obesity and multifaceted behavioral alterations in mice

**DOI:** 10.1186/s13229-022-00497-3

**Published:** 2022-05-10

**Authors:** Markus Wöhr, Wendy M. Fong, Justyna A. Janas, Moritz Mall, Christian Thome, Madhuri Vangipuram, Lingjun Meng, Thomas C. Südhof, Marius Wernig

**Affiliations:** 1 Department of Molecular and Cellular Physiology, School of Medicine, Stanford University, Stanford, CA 94305 USA; 2 Research Unit Brain and Cognition, Laboratory of Biological Psychology, Social and Affective Neuroscience Research Group, Faculty of Psychology and Educational Sciences, KU Leuven, 3000 Leuven, Belgium; 3 Leuven Brain Institute, KU Leuven, 3000 Leuven, Belgium; 4 Faculty of Psychology, Experimental and Biological Psychology, Behavioral Neuroscience, Philipps-University of Marburg, 35032 Marburg, Germany; 5 Center for Mind, Brain and Behavior, Philipps-University of Marburg, 35032 Marburg, Germany; 6 Departments of Pathology and Chemical and Systems Biology, School of Medicine, Institute for Stem Cell Biology and Regenerative Medicine, Stanford University, Stanford, CA 94305 USA; 7 School of Medicine, Howard Hughes Medical Institute, Stanford University, Stanford, CA 94305 USA; 8 Present Address: Cell Fate Engineering and Disease Modeling Group, German Cancer Research Center (DKFZ) and DKFZ-ZMBH Alliance, 69120 Heidelberg, Germany; 9 Present Address: HITBR Hector Institute for Translational Brain Research gGmbH, 69120 Heidelberg, Germany; 10 Present Address: Central Institute of Mental Health, Medical Faculty Mannheim, Heidelberg University, 68159 Mannheim, Germany

**Keywords:** Transcription factor, Autism, Obesity, Social behavior, Ultrasonic vocalization

## Abstract

**Background:**

The zinc finger domain containing transcription factor Myt1l is tightly associated with neuronal identity and is the only transcription factor known that is both neuron-specific and expressed in all neuronal subtypes. We identified Myt1l as a powerful reprogramming factor that, in combination with the proneural bHLH factor Ascl1, could induce neuronal fate in fibroblasts. Molecularly, we found it to repress many non-neuronal gene programs, explaining its supportive role to induce and safeguard neuronal identity in combination with proneural bHLH transcriptional activators. Moreover, human genetics studies found *MYT1L* mutations to cause intellectual disability and autism spectrum disorder often coupled with obesity.

**Methods:**

Here, we generated and characterized Myt1l-deficient mice. A comprehensive, longitudinal behavioral phenotyping approach was applied.

**Results:**

Myt1l was necessary for survival beyond 24 h but not for overall histological brain organization. *Myt1l* heterozygous mice became increasingly overweight and exhibited multifaceted behavioral alterations. In mouse pups, *Myt1l* haploinsufficiency caused mild alterations in early socio-affective communication through ultrasonic vocalizations. In adulthood, *Myt1l* heterozygous mice displayed hyperactivity due to impaired habituation learning. Motor performance was reduced in *Myt1l* heterozygous mice despite intact motor learning, possibly due to muscular hypotonia. While anxiety-related behavior was reduced, acoustic startle reactivity was enhanced, in line with higher sensitivity to loud sound. Finally, *Myt1l* haploinsufficiency had a negative impact on contextual fear memory retrieval, while cued fear memory retrieval appeared to be intact.

**Limitations:**

In future studies, additional phenotypes might be identified and a detailed characterization of direct reciprocal social interaction behavior might help to reveal effects of *Myt1l* haploinsufficiency on social behavior in juvenile and adult mice.

**Conclusions:**

Behavioral alterations in *Myt1l* haploinsufficient mice recapitulate several clinical phenotypes observed in humans carrying heterozygous *MYT1L* mutations and thus serve as an informative model of the human *MYT1L* syndrome.

**Supplementary Information:**

The online version contains supplementary material available at 10.1186/s13229-022-00497-3.

## Background

*MYT1L* has been proposed as a causative gene for intellectual disability (ID) and other phenotypes observed in cases with 2p25.3 deletions, such as autism spectrum disorder (ASD) and attention deficit hyperactivity disorder (ADHD) [1–4]. In the largest exome sequencing study to date, *MYT1L* was ranked among the prime candidate genes associated with ASD, clustering with other risk genes causing neurodevelopmental delay, such as *FOXP1* and *SETD5* [5]. Notably, the majority of these genes are involved in gene regulation, extending previous findings obtained in large human genetic studies [6–10]. For instance, in these studies, a de novo loss-of-function variant and a de novo likely damaging missense variant in the *MYT1L* gene were identified in two unrelated individuals with ASD from 2270 trios [7]. Case studies report that individuals carrying *MYT1L* mutations present with delays in the development of motor abilities, cognitive functions, and speech. Obesity and hyperphagia are common findings in these patients. Hyperactivity and aggressiveness are prevalent. In addition, hypotonia, motor deficits, sleep problems, and seizures occur in a significant number of cases. Dysmorphic features are rare, yet strabismus is often observed [1–4, 11–22]. Copy number variants affecting the *MYT1L* gene have also been implicated in schizophrenia (SCZ) [23–25]. Likewise, *MYT1L* gene polymorphisms were associated with SCZ [26]. A recent review on the role of *MYT1L* in neurodevelopmental disorders indicates that partial duplications are primarily associated with SCZ, while loss-of-function mutations are primarily associated with ID and ASD [27].

Myt1l is a member of the neural zinc finger (NZF) family of transcription factors, which contain Cys-Cys-His-Cys (C2HC) zinc finger motifs for DNA binding [28–30]. It is the only transcription factor known that is both specifically expressed in neurons and at the same time expressed in virtually all neuronal subtypes [30, 31]. Together with its two family members Myt1 and St18, its expression is induced soon after neurons are born from ventricular progenitor cells, but only Myt1l remains to be expressed into adulthood, whereas Myt1 and St18 are downregulated once neurons have matured [30, 31]. Myt1l is therefore tightly associated with the neuronal lineage and a candidate to maintain neuronal identity.

We previously identified Myt1l as a powerful transcription factor to induce morphological, biochemical, and functional neuronal properties in fibroblasts [32, 33]. In contrast to the proneural bHLH factor Ascl1, which was able to induce neuronal properties alone, Myt1l expression alone was not sufficient to induce neuronal genes in fibroblasts [34]. Of note, expression of only Ascl1 did successfully convert mouse fibroblasts into fully functional neurons, but many cells failed to silence mesodermal programs [35]. Genomic localization studies using chromatin immunoprecipitation (ChIP) and expression analyses revealed that Myt1l acts as a transcriptional repressor to suppress many non-neuronal programs including the mesodermal programs in fibroblasts infected with just Ascl1 [36]. This finding explains the cooperation of Ascl1 (a transcriptional activator and inducer of neuronal genes) and Myt1l to induce a faithful neuronal identity. These data further suggested that Myt1l might act to safeguard neuronal lineage identity by suppressing various non-neuronal programs, a mechanism exactly inverse of the transcriptional repressor REST, whose function is to suppress the neuronal program specifically [37]. One of the developmental pathways repressed by Myt1l is the Notch signaling pathway, a prominent pathway regulating neurogenesis [36]. Thus, despite the potential redundancy with its two closely related family members Myt1 and St18 during the early stages of neuronal differentiation, this finding could indicate a role during physiologic neuronal specification.

Although these studies have shed light into the molecular properties of Myt1l, its physiological function in the postnatal brain remains largely unexplored. Due to the association of *MYT1L* mutations with neurodevelopmental phenotypes in patients, we here sought to investigate the behavioral consequences of *Myt1l* haploinsufficiency in mice. While this paper was under review, a related study by Chen et al. was published, describing a similar mouse model that showed largely overlapping phenotypes [38].

## Materials and methods

### Animals and housing

Constitutive heterozygous *Myt1l*^+/-^ mutant mice were created by CRISPR/Cas9 targeting *Myt1l*. To generate the mutant mice, zygotes were isolated and electroporated with Cas9 proteins complexed with a guide sequence directed at exon 6, the first coding exon of the *Myt1l* gene. Transfected blastocysts were transferred into oviducts of day 0.5 pseudo-pregnant recipient ICR mice. Resulting pups were genotyped by Sanger sequencing of the PCR-amplified *Myt1l* region around the predicted cut site and several *Myt1l* indel mutations detected. We observed up to three different mutations in one animal suggesting continued Cas9 activity at least until the 4-cell stage. We focused on a *Myt1l* 7 base pair (bp) deletion allele because it is predicted to cause a frameshift and thus null allele. Mice containing this deletion 7 allele were bred with wild-type mice to achieve segregation of the various alleles and mice containing only wild-type and deletion 7 were obtained, which were used to start a colony. These *Myt1l*^+/-^ mice were maintained as heterozygotes on a C57BL6/N background strain by breeding to C57BL6/N (Charles River; Strain Code: 027) mice every 4–5 months. Experimental mice were bred and housed in facilities at the Lorry I. Lokey Stem Cell Research Building.

### Genotyping

**Fig. 1:**
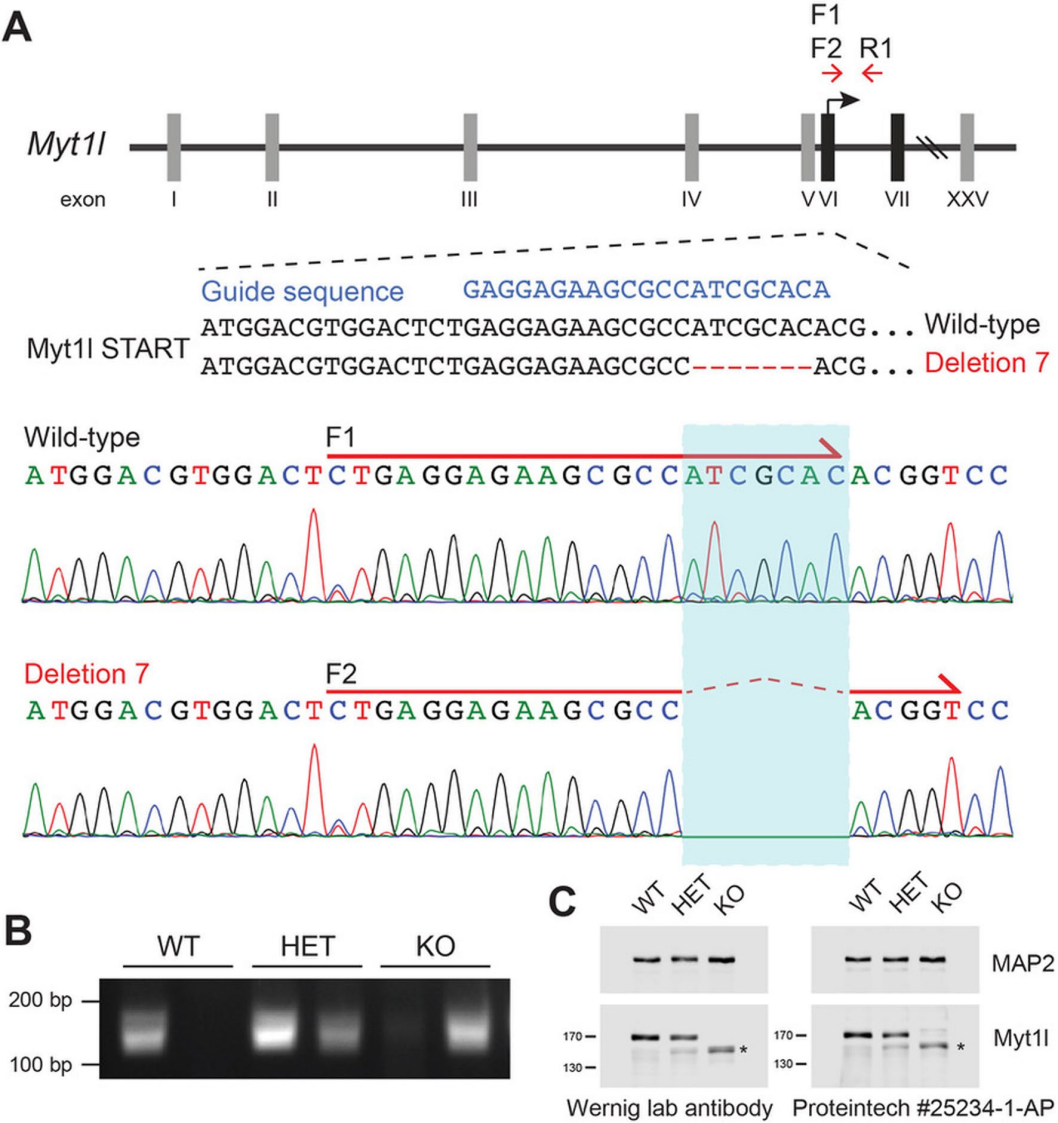
Generation of *Myt1l* mutant mice by CRISPR/Cas9 gene editing. **A** Schematic of the gene editing strategy. The guide RNA sequence (blue text) targets seven base pairs downstream of the translation start site in exon VI of the mouse *Myt1l* locus and generates a frameshift deletion. The two forward and one reverse primers (red arrows) used for genotyping are noted. The forward primers, F1 and F2, incorporate the presence and absence of the seven base pairs, respectively. **B** PCR genotyping of *Myt1l* mutant mice. As expected, the primer pair F1 and R1 (left lanes) shows a PCR amplification of only wild-type alleles, whereas the primer pair F2 and R1 (right lanes) amplifies only the deletion allele, allowing unequivocal genotyping of wild-type *Myt1l*^+*/*+^, heterozygous *Myt1l*^+/-^*,* and homozygous *Myt1l*^*−/−*^ mice. (**C**) Immunoblotting of Myt1l in WT, HET, and KO E18.5 brains. Whole brain lysates were subjected to Western blotting using two different Myt1l antibodies with similar results. MAP2 was used as a loading control. Note with both antibodies an extra band(*) appeared in mice carrying the mutant allele corresponding to a Myt1l-related protein of ~ 158 kDa

PCR to detect *Myt1l* frameshift deletion was performed with 50–200 ng of DNA extracted with QuickExtract DNA Extraction Solution (Lucigen, Middleton, WI, USA) from tail biopsies, using Taq polymerase in the Bio-Rad T100 Thermal Cycler. PCR for *Myt1l* deletion was performed using a forward primer for the wild-type allele F1, a separate forward primer that incorporates the deletion allele F2, and a common reverse primer R1 (see also Fig. 1A). The primers were as follows: *Myt1l* wild-type forward *CTG AGG AGA AGC GCC ATC GCA*; *Myt1l* deletion forward *CTG AGG AGA AGC GCC ACG GT*; and reverse *CAC TGG TAC TCT TCT TCC ACG GAA AAT TAC C*.

### Breeding scheme for embryonic neurobiological characterization

Neurobiological effects of *Myt1l* deletion were assessed by comparing male and female constitutive homozygous *Myt1l*^*−/−*^ mutant mice to wild-type *Myt1l*^+*/*+^ and heterozygous *Myt1l*^+/-^ littermate controls. To generate the experimental cohort, pairs of male and female heterozygous *Myt1l*^+/-^ mice were set up in the evening and females were checked for plugs the following morning.

### Dissection and immunoblotting

Whole mouse brains were dissected from E18.5 mice. Tissue was homogenized in ice-cold cell lysis buffer consisting of 0.5% Tween-20, 50 mM Tris pH 7.5, 2 mM EDTA, and 1 mM DTT with protease inhibitors. The lysate was incubated on ice for 15 min. Nuclei were pelleted by centrifugation at 3200 rpm for 1 min at 4 °C, and the pellet was resuspended in NP-40 lysis buffer consisting of 0.5% NP-40, 50 mM Tris pH 8.0, 150 mM NaCl, 2 mM EDTA, and 1 mM DTT with protease inhibitors. Samples were spun at 14,000 rpm for 10 min at 4 °C, and the supernatant was pipetted into a new tube. Western blot was run as described [39].

### qRT-PCR

RNA was isolated using Trizol (Invitrogen) and RNA Clean & Concentrator (Zymo) and reverse-transcribed with Superscript III (Invitrogen). mRNA levels were quantified by real-time PCR assay using SYBRGreen (Thermo Fisher Scientific) and the Applied Biosystems QuantStudio 7 Pro Real-Time PCR system. Expression values were expressed as percent of MAP2 using the formula: 2-CT (target mRNA)/2-CT (housekeeping mRNA) × 100. Primers used: MAP2-F: CGGTCTCCAGGGATGAAGTG; MAP2-R: ACTTGCTGCTGTGGTTTTCC; Myt1l-F: ATGTTCCCACAACCACACCA; Myt1l-R: TACCGCTTGGCATCGTCATA.

### Antibodies

The following antibodies were used for western blot: rabbit anti-Myt1l (1:500; Millipore ABE2915) and mouse anti-β-Actin (1:10,000; Sigma A5441). Rabbit anti-Tbr2 (1:500; ab23345), rat anti-Ctip2 (1:500; ab18465), and rabbit anti-Sox2 (1:250; Millipore AB5603) were used for immunofluorescence.

### Histology in embryonic tissue

The morning the plug was observed was designated embryonic day 0.5. Embryos from deeply anesthetized pregnant dams were collected at E15.5 or E18.5. Uterine horns were placed in ice-cold phosphate-buffered saline (PBS). The heads of the embryos were drop-fixed in 4% paraformaldehyde (PFA) for 3 h, cryoprotected in 30% sucrose overnight, fresh frozen in powdered dry ice, embedded in O.C.T. Compound (VWR), and stored at − 80 °C until use. 40 µm free-floating coronal sections were cut using Leica CM3050 S into 1X phosphate buffer (P.B.) containing 0.02% sodium azide. Thionin staining was performed as described [40]. For immunofluorescence, sections were incubated in 10 mM sodium citrate, pH 6.0 for 10 min at 85 °C for antigen retrieval. They were washed three times with 1X PBS and then blocked for 1 h with 1X PBS containing 0.3% Triton X-100 (Tx-100) and 5% normal goat serum (NGS). Sections were incubated with primary antibody overnight at 4 °C. On the following day, after three washes in 1X PBS, sections were incubated in the appropriate Alexa Fluor secondary antibodies (Molecular Probes) diluted 1:1,000 in blocking solution for 2 h at room temperature. The sections were counterstained with DAPI in PBS for 10 min, washed three times in 1X PBS, mounted on slides, and cover-slipped (Antifade Gold, Life Technologies).

### Quantitative image analysis

Brain anatomy and immunofluorescent labeling were assessed using three mouse triplets (*Myt1l*^+*/*+^, *Myt1l*^+/-^, and *Myt1*^*−/−*^) from three different litters. Nissl staining embryos at age E18.5 were analyzed in slices corresponding to sections 125 (rostral) and 155 (caudal) of the Allen Atlas of the Developing Mouse Brain. Images were taken on an Olympus Slide Scanner VS200 using the 20 × objective. In the rostral section, we measured the diameter of the cortical plates (CP) at motor cortex and barrel cortex positions, the diameter of the corpus callosum, as well as the area of striatum and septum. In the caudal section, we measured the diameter of the pallium at motor cortex and barrel cortex positions, the diameter of the hippocampus from the alveus-CA1-dentate-gyrus, as well as the area of thalamus and hypothalamus.

Immunofluorescence was imaged using a Nikon A1R laser scanning confocal and 40 × oil immersion objectives. Sox2 expression was assessed by measuring the area of the Sox2-positive signal in a 100 × 100 µm square region of the ventricular zone (VZ) (age E15.5, primary motor cortex). The borders of the VZ and CP were determined using the Sox2 and DAPI signals. Trb2 expression and Ctip2 expression were used to measure the diameters of the proximal VZ (Trb2-negative), the distal VZ (Tbr2-positive and Tbr2-negative cells), the multipolar cell accumulation zone (MAZ, part of subventricular zone, all cells Tbr2-positive), the distal subventricular zone (some isolated Tbr2-positive cells), the intermediate zone (no Tbr2 signal, no/weak Ctip2 signal), the CP (band of strong Ctip2 signal), and the marginal zone. Ctip2 positive cells were counted in a 50 × 100 µm rectangle in the CP.

### General overview on behavioral phenotyping

Behavioral effects of *Myt1l* haploinsufficiency were tested by comparing male and female constitutive heterozygous *Myt1l*^+/-^ mutant mice to wild-type *Myt1l*^+*/*+^ littermate controls. C57BL6/N dams were bred to *Myt1l*^+/-^ sires to generate the experimental cohort. The day of birth was defined as postnatal day (PND) 0. In order to avoid litter effects, only litters with both genotypes were included in the experiments. After weaning on PND 21, same-sex littermates of mixed genotypes were socially housed in groups of 2–5 mice in individually ventilated cages with corn cob bedding (Universal Euro II Type Long; 522.6 cm^2^ floor space, 5653.5 cm^3^ living space, 12.7 cm height; Innocage; Innovive, San Diego, CA, USA). Mice were maintained on a 12-h light/dark cycle and were provided food (irradiated laboratory animal diet, 18% protein; Teklad; Madison, WI, USA) and water ad libitum. Mice were identified by paw tattoo, using non-toxic animal tattoo ink (Ketchum Green Animal Tattoo Ink Paste, Ketchum Manufacturing Inc., Brockville, Canada). The ink was gently inserted subcutaneously through a 30 gauge hypodermic needle tip into the center of the paw at PND 10. Mouse tail snips for genotyping were collected by dissecting ~ 0.3 cm of tail the day of tattooing.

A comprehensive, longitudinal behavioral phenotyping approach was applied, including behavioral assays relevant to all human ASD core symptoms [41–43]. For behavioral phenotyping, mice of N = 7 litters (8 ± 0.38 pups/litter; mean ± SEM) were tested for sensory and motor abilities as well as isolation-induced ultrasonic vocalizations in the homing test at PND 10. After weaning at PND 21, they were tested in a battery of behavioral assays in the following order: Activity box, open field, elevated plus maze, Y-maze, social approach in the three-chamber assay, direct reciprocal social interaction, accelerated rotarod, nest building, and repetitive behavior were performed at the age of 2–4 months. Spatial learning and reversal learning, acoustic startle and pre-pulse inhibition of acoustic startle, and fear conditioning were performed at the age of 6–12 months. Mice of the behavioral cohort were also used for determining the body weight gain trajectory. Body weight was measured after every behavioral assay and every 3 weeks for up to 1 year starting from PND 42. Body temperature was measured at 14 months. IPTT-300 transponders (Bio Medic Data Systems, Seaford, Delaware, USA) were injected subcutaneously and longitudinally above the shoulder. Mice were monitored for infection and allowed to recover over 3 weeks, after which the body temperature was read with the IPTT 5515 handheld reader by holding it over a freely moving mouse (DAS-7007S; Bio Medic Data Systems, Seaford, Delaware, USA). The behavioral cohort consisted of *N* = 30 *Myt1l*^+/-^ mice (females: *N* = 12; males: *N* = 18) and *N* = 26 *Myt1l*^+*/*+^ littermate controls (females: *N* = 9; males: *N* = 17).

All behavioral experiments were carried out between 7 am and 7 pm during the light phase of the 12-h light/dark cycle. All behavioral assays were conducted and analyzed blind to genotype.

### Homing test and isolation-induced ultrasonic vocalizations

For assessing sensory and motor abilities as well as isolation-induced ultrasonic vocalizations, pups were isolated from their mother and littermates for 10 min at room temperature (21–23 °C), using a modified protocol [44]. Individual pups were transferred to a cage with corn cob bedding (Universal Euro II Type Long; Innocage; Innovive). A new cage with clean bedding was used for each test to avoid olfactory cues. Soiled corn cob bedding from the home cage was evenly spread on one side (1/3 of the cage, nest area), while the rest of the cage was covered with clean bedding (2/3 of the cage, clean area). The soiled zone was counterbalanced across individual mouse pups. The pup was placed in the middle of the cage and video-recorded for 10 min. The floor of the arena was virtually subdivided into three zones, i.e., soiled nest zone, clean center zone, and clean no nest zone, to allow behavioral scoring using The Observer XT 11 software (Noldus, Wageningen, The Netherlands). Behavioral scoring included locomotor activity by counting line crossings. Homing performance was scored as latency to reach and the time spent in the soiled nest zone. Emission of isolation-induced ultrasonic vocalizations was monitored by an UltraSoundGate Condenser Microphone CM16 sensitive to frequencies of 15–180 kHz (flat frequency response between 25 and 140 kHz; ± 6 dB; Avisoft Bioacoustics, Berlin, Germany). The microphone was placed in the roof of the cage lid, ~ 12 cm above the floor. The microphone was connected via an UltraSoundGate 416 USG audio device (Avisoft Bioacoustics) to a computer, where acoustic data were recorded with a sampling rate of 250,000 Hz (16 bit) by Avisoft RECORDER (version 2.97). For acoustic analysis, recordings were transferred to Avisoft SASLab Pro (version 5.2.12) and a fast Fourier transform was conducted (512 FFT length, 100% frame, Hamming window, and 75% time window overlap), resulting in spectrograms with 488 Hz of frequency and 0.512 ms of time resolution. Call detection was provided by an automatic threshold-based algorithm (amplitude threshold -65 dB; hold time 10 ms; high-pass filter 30 kHz). Accuracy of call detection was verified by an experienced user. When necessary, missed calls were marked manually to be included in the automatic parameter analysis. Total numbers of isolation-induced ultrasonic vocalizations were calculated for the entire session and in 60 s time bins to visualize the time course of the ultrasonic vocalization response. Additional parameters included latency to start calling, call duration, peak frequency, peak amplitude, and frequency modulation. Finally, the temporal organization of isolation-induced ultrasonic vocalizations emission was assessed through sequential analyses and call subtypes were determined by means of density plots. After the 10-min isolation period, body temperature and weight were determined. For body temperature determination, a Testo 110 thermometer with surface sensor (Testo AG, Lenzkirch, Germany) was used. Body weight was measured using a palmscale (PS7-200; precision: 0.01 g; MyWeigh Europe, Hückelhoven, Germany).

### Activity box

Locomotor activity, exploratory behavior, and anxiety-related behavior were assessed under direct white light (~ 20 lx) in an activity box (ENV-510, 27.31 × 27.31 × 20.32 cm; Med Associates, Fairfax, VT, USA), using a modified protocol [45]. The activity box was housed within a sound-attenuating chamber, equipped with a ventilation fan and illuminated by a single overhead light. Mice were allowed to freely explore the activity box on two consecutive days for 30 min each day. The position of the mouse within the arena was tracked in three dimensions by arrays of infrared light beams connected to a computer running Activity Monitor software (Med Associates, Fairfax, VT, USA). This software was used to calculate distance traveled and the number of rearings during 1-min time bins, which were summed together to calculate total values throughout the entire 30-min test session. The activity box was thoroughly cleaned between each mouse using 70% ethanol to avoid olfactory cues.

### Open field

Locomotor activity, exploratory behavior, and anxiety-related behavior were also measured under more anxiogenic conditions under direct bright white light (~ 200 lx) in a white open field (34 × 34 × 40 cm), using a modified protocol [46]. At the beginning of the test, individual mice were placed into one corner of the open field. Mice were allowed to freely explore the open field for 10 min. Distance traveled and time spent in the center were recorded and analyzed using Viewer III tracking software (Biobserve, Bonn, Germany). The center area was defined as the 28 × 28 cm central section of the open field. Fecal boli were counted at the end of test session. The open field was thoroughly cleaned between each mouse using 70% ethanol to avoid olfactory cues.

### Elevated plus maze

Anxiety-related behavior in the elevated plus maze was measured under indirect white light (~ 50 lx), using a modified protocol [46]. The gray maze was elevated 50 cm above the floor and consisted of four arms, i.e., two open arms and two closed arms with 15-cm-high walls, each arm measuring 35 cm long and 5 cm wide. Individual mice were initially placed in the center of the maze, facing an open arm. Mice were then allowed to freely explore the maze for 5 min. The amount of time spent in each arm, number of entries into each arm, total distance traveled, and average velocity were recorded and analyzed using Viewer III tracking software (Biobserve, Bonn, Germany). The elevated plus maze was thoroughly cleaned between each mouse using 70% ethanol to avoid olfactory cues.

### Y-maze

Spatial working memory in the Y-maze was measured under indirect red light conditions, using a modified protocol [46]. A gray plastic Y-maze was used to evaluate spontaneous alternations reflecting spatial working memory. The maze consisted of three arms that were spaced 120° angle from each other (dimensions of each arm 40 × 10 × 17 cm). Mice were individually placed in the distal end of one arm and allowed to freely explore the entire maze for 10 min. A completed arm entry was defined as the entering of the mouse with all four limbs. The sequence of arm entries was recorded and analyzed using the Viewer III tracking system (Biobserve, Bonn, Germany). Visiting all three different arms consecutively was termed a ‘correct’ triad, and visiting one arm twice in three consecutive entries was termed a ‘wrong’ triad. Correct alternation percentage was calculated using the following formula: %Alternation = (Number of Alternations/[Total number of arm entries − 2]) × 100. The Y-maze was thoroughly cleaned between each mouse using 70% ethanol to avoid olfactory cues.

### Social approach assay

Social motivation was evaluated in a three-chamber box (60 × 30 × 30 cm^3^) made of transparent polycarbonate under indirect red light conditions, using a modified protocol [47]. Retractable doorways built into the two dividing walls controlled access to the side chambers. The test session began with a 10-min habituation session during which lack of an innate side preference was confirmed. The subject mouse was then briefly confined to the center chamber, while a clean novel object, an empty metal enclosure, was placed in one of the side chambers. A novel stimulus mouse previously habituated to the enclosure was placed in an identical metal enclosure located in the other side chamber. The side containing the novel object and the novel stimulus mouse alternated between the left and right chambers across subject mice. After both stimuli were positioned, the two side doors were simultaneously lifted and the subject mouse was allowed access to all three chambers for 10 min. C57BL6/N mice served as stimulus mice. Stimulus mice were age-matched and of the same sex as the subject mouse. The amount of time spent exploring the metal enclosures, the amount of time spent in each chamber, number of entries into each chamber, and total distance traveled were recorded and analyzed using Viewer III tracking software (Biobserve, Bonn, Germany). The three-chamber box and the metal enclosures were thoroughly cleaned between each mouse using 70% ethanol to avoid olfactory cues.

### Interaction-induced ultrasonic vocalizations

Emission of interaction-induced ultrasonic vocalizations was assessed during direct reciprocal social interaction in a test chamber (50 × 25 × 30 cm^3^) made of transparent polycarbonate under indirect red light conditions, using a modified protocol [48]. A transparent polycarbonate lid containing 16 holes (diameter 1.3 cm) was placed on the top of the social interaction chamber to reduce background noise. Clean corn cob bedding was evenly spread on the floor. Male–female and female–female pairs of mice were allowed to socially interact for 5 min after the subject mouse was habituated to the test environment for 1 min. Age-matched C57BL6/N mice served as stimulus mice. Stimulus mice were tail-marked. Interaction-induced ultrasonic vocalizations emitted by pairs were monitored by an UltraSoundGate Condenser Microphone CM16 (Avisoft Bioacoustics) placed in the roof of the lid, ~ 30 cm above the floor. The microphone was connected via an UltraSoundGate 416 USG audio device (Avisoft Bioacoustics) to a computer, where acoustic data were recorded with a sampling rate of 250,000 Hz (16 bit) by Avisoft RECORDER (version 2.97). For acoustic analysis, recordings were transferred to Avisoft SASLab Pro (version 5.2.12) and a fast Fourier transform was conducted (512 FFT length, 100% frame, Hamming window, and 75% time window overlap), resulting in spectrograms with 488 Hz of frequency and 0.512 ms of time resolution. An experienced user counted the number of ultrasonic vocalizations in 1-min time bins. The social interaction chamber was thoroughly cleaned between each pair of mice using 70% ethanol to avoid olfactory cues.

### Accelerated rotarod

Motor coordination and motor learning was tested under white room light using a five-station rotarod treadmill (ENV-575 M, Rota-Rod software; Med Associates, Fairfax, VT, USA), as previously described [45]. Testing consisted of three trials per day, separated by at least 60 min each, over the course of 4 days, i.e., 12 trials in total. On the first day of testing, mice were acclimated to the apparatus by placement on the stationary rotarod for 30 s. Two versions of the rotarod task were used. First, the standard task with an accelerating rod from 4 to 40 rpm within 300 s was applied for 2 days, i.e., 6 trials in total. Then, the standard range of acceleration from 4 to 40 rpm was expanded using custom hardware purchased from the vendor, allowing us to test an acceleration of 8–80 rpm, while maintaining a constant rate of acceleration over 300 s. The version with an acceleration of 8–80 rpm was also applied for 2 days, i.e., 6 trials in total. In both versions, the latency to fall off and the latency to make one complete revolution while hanging on were measured. A trial was stopped after 300 s (maximum speed, no further acceleration). The rotarod was thoroughly cleaned between each trial using 70% ethanol to avoid olfactory cues.

### Nest building

Nest building was measured under white room light, using a modified protocol [49]. After mice were habituated for 15 min to a novel cage with corn cob bedding but no other nest material (Universal Euro II Type Long; Innocage; Innovive), a 5 × 5 cm square of pressed cotton (Nestlet; Ancare, Bellmore, NY) was placed in a random cage corner, and the net increase in nest width and nest height was measured after 30, 60, and 90 min. At the end, nest quality was scored.

### Repetitive behavior

Repetitive behavior was measured under indirect white light (~ 40 lx), using a modified protocol [49]. After mice were habituated for 10 min to a novel cage with corn cob bedding but no other nest material (Universal Euro II Type Long; Innocage; Innovive), self-grooming of all body regions was recorded for 10 min from the side (Brio 4 K ultra HD webcam; Logitech Europe S.A.) and analyzed by a trained observer using The Observer XT 11 software (Noldus).

### Spatial learning and reversal learning

Spatial learning and reversal learning were assessed in a Barnes maze under direct bright white light (~ 200 lx), using a modified protocol [50]. The white maze consisted of a brightly lit circular open platform (92 cm diameter) with 20 equally spaced holes (5 cm diameter) along the perimeter. Underneath the designated target hole, an escape box (7 cm deep, 7 cm width, and 10 cm length) was placed. Underneath the remaining holes, false escape boxes were placed, made of the same material as the escape box. Extra-maze cues were placed on the surrounding walls to serve as reference cues to learn the position of the target escape hole. At the beginning of the test, individual mice were placed in the center of the maze in a holding chamber (15 × 15 cm) for 30 s. Once the chamber was lifted, the mice were allowed to freely explore the maze for 90 s with 19 of the 20 holes closed and were assayed for their ability to spatially navigate the maze to find the target escape hole. The target escape box was positioned underneath the maze as a small, dark, recessed chamber, which the mice naturally sought out, taking advantage of their desire to escape brightly lit and exposed environments. During the initial, consecutive four-day training period, the mice learned the spatial location of the target hole, with four trials conducted per day (~ 2 h inter-trial interval). For reversal learning, the target hole was rotated by ~ 180°. The mice were again trained for four consecutive days, with four trials conducted per day (~ 2 h inter-trial interval). The maze was thoroughly cleaned between each trial using 70% ethanol to avoid olfactory cues. Data acquisition and analysis were performed using the Viewer III tracking system (Biobserve, Bonn, Germany). During spatial learning and reversal learning, the latency needed to reach the target hole was measured. During the last spatial learning day, the number of visits of the target hole and adjacent holes was quantified and used as a preference measure. Similarly, the number of visits of the initial target hole, the reversal target hole, and adjacent holes was quantified during the first reversal learning day. Affinity was determined by measuring the time spent within the initial target hole and the time spent within the reversal target hole. For hole visit and affinity, an entry was defined as the mouse entering the hole with the snout or major parts of the body. Primary errors were also calculated as entering the wrong hole before reaching the target hole.

### Acoustic startle reactivity and pre-pulse inhibition of acoustic startle

Acoustic startle reactivity and pre-pulse inhibition of acoustic startle were measured using the Kinder Scientific startle reflex system (Kinder Scientific, Poway, CA, USA), as previously described [46]. Data were analyzed with the Startle Monitor II software (Kinder Scientific). Mice were individually placed in a small cage atop a force plate within a sound attenuation chamber without light. Background noise was set at 65 decibel (dB). The startle response, defined as the change in amplitude of force in response to an unexpected acoustic stimulus, was measured. The peak values of the absolute force mice placed on the bottom of the cage were measured as the startle response. For the acoustic startle reactivity experiment, 50 ms noise at 75, 85, 95, 105, and 115 dB was presented. Each stimulus was repeated 10 times. For the pre-pulse inhibition experiments, 50 ms noise at 115 dB was presented, with preceding noise at 0, 68, 71, or 77 dB. The acoustic startle reactivity experiment had three phases. First, the startle response was determined by presenting 10 consecutive 115 dB pulse trials. The following trials were then presented 10 times each in pseudorandom order: 115 dB pulse with 0 dB pre-pulse, 68 dB pre-pulse, 71 dB pre-pulse, and 77 dB pre-pulse. The 115 dB pulse followed each pre-pulse at a 100-ms onset–onset interval. Then, the startle response was again determined by presenting 10 consecutive 115 dB pulse trials. The percent inhibition of the startle amplitude displayed during pulse trials was calculated for each pre-pulse/ pulse pair. For both experiments, mice were given 3 min of habituation time before the sound was delivered. Stimulus sequence and inter-stimulus intervals were both pseudo-randomized. The cage was thoroughly cleaned between each trial using 70% ethanol to avoid olfactory cues.

### Fear conditioning

Fear conditioning was conducted using the Coulbourn fear conditioning system (Coulbourn Instruments, Holliston, MA, USA), as previously described [46]. Data were analyzed with the FreezeFrame software (Actimetrics Software, Wilmette, IL, USA). On the training day, individual mice were placed in the fear conditioning chamber (18.5 × 18.5 × 21.5 cm; H10-11 M-TC; Coulbourn Instruments) outfitted with a metal grid floor and located in the center of a sound-attenuating cubicle with white house light (~ 20 lx; Coulbourn Instruments). The conditioning chamber was cleaned using 70% ethanol to provide background odor. A ventilation fan provided background noise at ~ 55 dB. As the conditioned stimulus (CS), a 2 kHz tone was presented at 85 dB for 30 s. As the unconditioned stimulus (US), a 0.75 mA foot shock was applied for 2 s through the Coulbourn precision animal shocker (Coulbourn Instruments). The foot shock co-terminated with the tone. After a 2-min habituation period, three CS–US pairings separated by 1-min inter-stimulus intervals (ISI) were delivered. Mice remained in the conditioning chamber for another 60 s before being returned to their home cages. In the context test, 24 h after training, mice were placed back into the original conditioning chamber for 5 min to assess contextual recall. The conditioning chamber was thoroughly cleaned between each mouse using 70% ethanol to avoid olfactory cues. During the altered context test, 48 h after training, the conditioning chamber was modified by changing its metal grid floor to a plastic sheet, white metal side walls to plastic walls decorated with stripes of various colors, and the background odor of 70% ethanol to 1% vanilla. Mice were placed in the altered chamber for 5 min. After this 5-min period, the CS was delivered for 1 min to assess cued recall. Freezing behavior was defined as the absence of motion lasting 1 s or longer was recorded and analyzed automatically in 30-s time bins using the FreezeFrame software.

### Statistical analysis

Statistical analysis was performed using SPSS (IBM Statistics SPSS, version 26, Chicago, IL, USA) and GraphPad Prism (GraphPad Software, version 9.1.0, San Diego, CA, USA). SigmaPlot (Systat Software GmbH, version 13, Erkrath, Germany), BioRender, Adobe Creative Suite 6 Photoshop and Illustrator were used to create figures. The comparison between the observed genotype distribution and expected Mendelian distribution was assessed by the chi-square goodness-of-fit test. A significant trend among the survival curves was assessed using a logrank Mantel–Cox test. For the other statistical comparisons, primarily analyses of variance (ANOVAs) were used. Specifically, to compare developmental profiles and behavioral phenotypes, ANOVAs with the between-subject factors genotype and sex or ANOVAs for repeated measurements with the between-subject factors genotype and sex and relevant within-subject factors, such as age, time, trial, or preference, were conducted. ANOVAs were followed by *post hoc* testing using individual ANOVAs or paired and unpaired *t* tests when appropriate. A *p* < 0.050 was considered statistically significant.

## Results

### Generation of Myt1l mutant mice

We generated a predicted loss-of-function allele of *Myt1l* in mice (Fig. 1A) by electroporating zygotes with CRISPR/Cas9 proteins pre-complexed with guide RNAs targeting exon 6, the first coding exon of the *Myt1l* gene, as described [51]. Resulting newborn pups were genotyped and showed different indel mutations. One of them was a 7 bp deletion in exon 6 leading to a frameshift mutation at c.28 of the *Myt1l* coding sequence resulting in predicted stop codon at c.231 (resulting in 77 amino acids). Mice containing this allele were bred with wild-type mice to establish *Myt1l*^+/-^ mice, which were viable and fertile. When mice heterozygous for this mutation were interbred, we obtained newborn mice of all three genotypes (albeit not at expected ratios, see below) as determined by PCR analysis using wild-type and deletion-specific primers (Fig. 1B). When brains from mice with the three different genotypes were analyzed by Western blot analysis, we could confirm that the full-length, ~ 170 kDa Myt1l band present in wild-type *Myt1l*^+*/*+^ mice was absent in homozygous *Myt1l*^*−/−*^ mice and decreased in heterozygous *Myt1l*^+/-^ mice by about 25% (Fig. 1C). Quantitative immunoblotting showed that the intensity of the Myt1l band is decreased in *Myt1l*^+/-^ mice (Additional file 1: Figure S1A, B). As expected, the 7 bp deletion did not affect the levels of Myt1l mRNA produced by neurons (Additional file 1: Figure S1C). Surprisingly, however, we detected a band stained by two independent Myt1l antibodies corresponding to a ~ 158 kDa protein that appeared in mutant cells (Fig. 1C). Since our deletion produces a frame shift, the variant protein must be generated from an alternative start codon. There happens to be another Methionine codon about 99 amino acids downstream of the known start codon, which may be utilized as translational initiation in the mutant mRNA resulting in a protein of about ~ 158 kDa.

### Myt1l is essential for postnatal survival

**Fig. 2:**
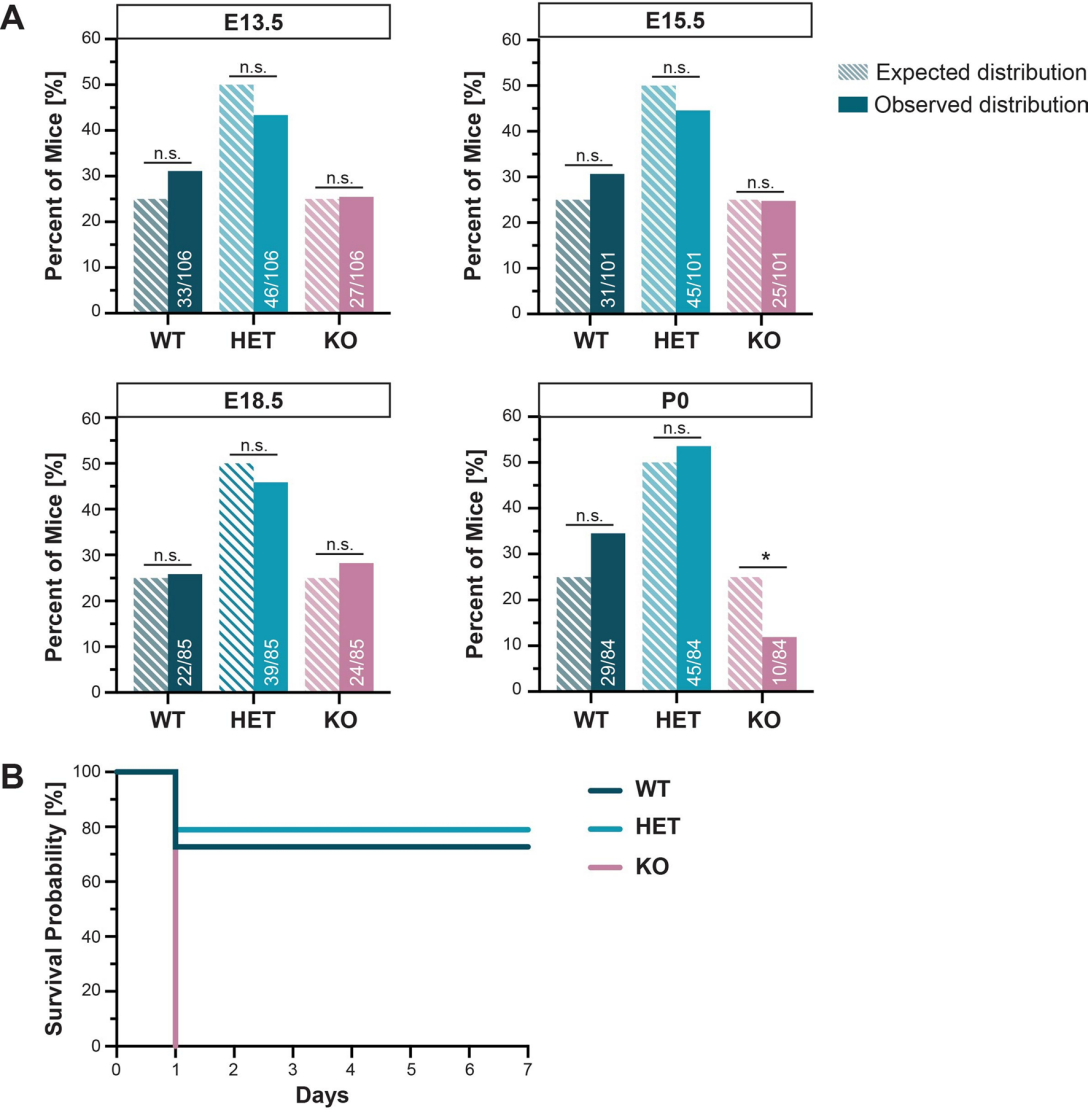
Myt1l is essential for postnatal survival. **A** Genotype distribution of *Myt1l* offspring at various ages. The dashed bars represent the expected percentage of mice for a genotype, assuming Mendelian inheritance from two heterozygous parents. The solid bars show the observed distribution based on 10–13 litters. Approximately 25% of mice were WT, 50% HET, and 25% KO at ages E13.5, E15.5, and E18.5. In contrast, only 12% of KO were present at PND 0. Numbers in bars represent number of mice per genotype/total number of mice. The chi-square test indicated a significant difference between the expected and observed distribution of KO pups at PND 0 (*p* = 0.011). n.s. = not significant; *p < 0.05. **B** Kaplan–Meier survival curve comparing the three genotypes after birth. WT and HET survive past PND 7, while all *Myt1l* KO die by PND 1. A logrank Mantel–Cox test showed a significant trend for the survival probability of KO pups (*p* = 0.0019)

While *Myt1l*^*−/−*^ embryos were present at the approximate expected Mendelian ratios up to embryonic age 18.5 (E18.5) (WT: 26%, N = 22; HET: 46%, N = 39; KO: 28%, N = 24), they fell significantly below 25% at postnatal day (PND) 0 (WT: 34%, N = 29; HET: 54%, N = 45; KO: 12%, N = 10) (Fig. 2A). Although neonatal *Myt1l*^*−/−*^ mice appeared normal, any surviving *Myt1l*^*−/−*^ mouse pups died within 24 h after birth (Fig. 2B), showing that Myt1l is essential for postnatal survival.

### Myt1l-deficient mice exhibit overall normal neurogenesis and brain structure

**Fig. 3:**
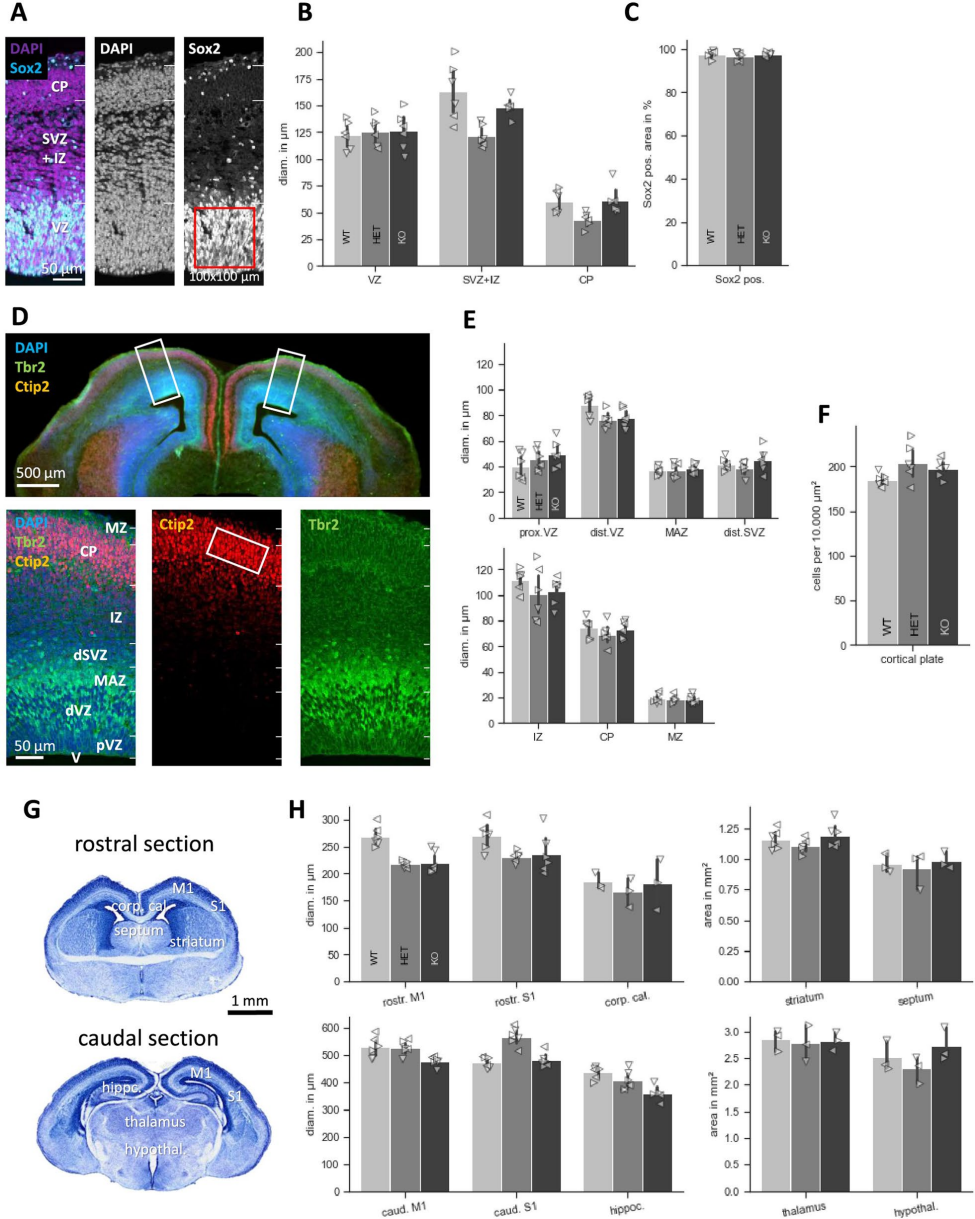
No overt anatomical defects in the developing mouse brain of *Myt1l* mutant mice. **A**, **B** Sox2 staining (cyan) in E15.5 Myt1l (*N* = 3/genotype) shows no gross difference in VZ compared with littermate controls. **C** The number of Sox2-positive neurons was quantified as the percentage of Sox2-positive area in a 100 × 100 µm area in the ventricular zone. **D** Coronal sections stained for Tbr2 (green), Ctip2 (red), and counterstained with the nuclear stain DAPI (blue). **E** The size of layers expressing Tbr2 and Ctip2 remains similar between all three genotypes (*N* = 3/genotype). **F** The density of Ctip2 positive neurons was assessed by counting in a field of 50 × 100 µm in the central CP. **G** Nissl-stained whole brains from E18.5 embryos. **H** *Myt1l* mutants show similar gross brain anatomy, including the rostral and caudal cortex (motor cortex region M1, sensory cortex barrel region S1), corpus callosum, striatum, septum, hippocampus, thalamus, and hypothalamus, compared to WT and HET. *N* = 3/genotype. (Left and right hemispheres of one animal are indicated as arrowheads leading in the same direction; MZ marginal zone, CP cortical plate, IZ intermediate zone, (d)SVZ (distal) subventricular zone, MAZ multipolar cell accumulation zone, (p)VZ (proximal) ventricular zone, V ventricle)

Given Myt1l’s involvement in neuronal specification by reprogramming and in particular its ability to repress neural progenitor programs contributing to neuronal differentiation, we characterized cortical neurogenesis of E15.5 midgestation embryos. We stained for Sox2 to label VZ progenitor cells, for Tbr2 to label basal progenitor cells and Ctip2 to label postmitotic neurons in the developing CP (Fig. 3A, D). Qualitative epifluorescence microscopy initially appeared to show an enhanced Tbr2 signal, but quantitation of confocal microscopic sections showed no significant changes between mutant and wild-type brains (Fig. 3A–F). Consistent with the apparently normal neurogenesis, *Myt1l* mutant mice did not exhibit any histopathological abnormalities following Nissl staining of E18.5 serial brain sections (Fig. 3G, H; Additional file 2: Figure S2).

### Intact maternal odor preference in the homing test

**Fig. 4:**
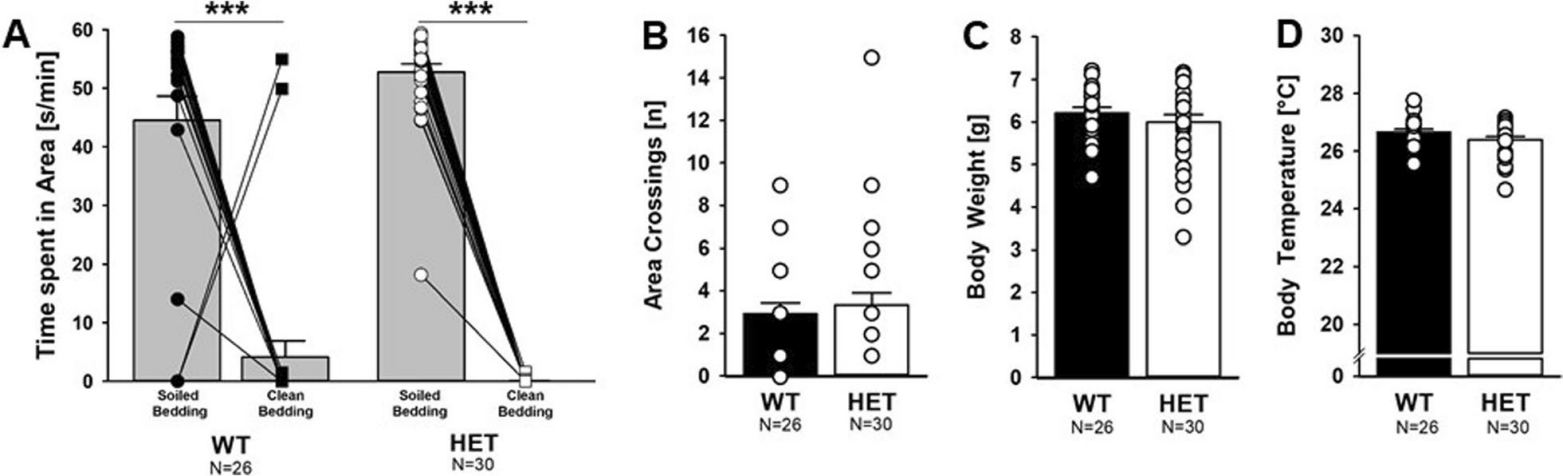
Effects of *Myt1l* haploinsufficiency on maternal odor preference in the homing test. **A** Maternal odor preference is intact in *Myt1l*^+/-^ mouse pups. Repeated-measures ANOVA with the between-subject factors genotype (G) and sex (S) and the within-subject factor preference (P). P: F_1,52_ = 214.668; *p* < .001; all other *p* > 0.050. **B** Area crossings are unchanged in *Myt1l*^+/-^ mouse pups. ANOVA with the between-subject factors genotype (G) and sex (S); all *p* > 0.050 **C** Body weight is unchanged in *Myt1l*^+/-^ mouse pups. ANOVA with the between-subject factors genotype (G) and sex (S); all *p* > 0.050. **D** Body temperature is not altered in *Myt1l*^+/-^ mouse pups. ANOVA with the between-subject factors genotype (G) and sex (S); all *p* > 0.050. All data are means ± SEM, combined across males and females; * p < 0.05; **p < 0.01; ***p < 0.001

Next, we performed a comprehensive behavioral characterization (see Additional file 6: Table S1 for a detailed overview on the results of the statistical analyses). We first applied the homing test and asked whether *Myt1l* haploinsufficiency affects the preference that mouse pups typically display for the area with soiled bedding from the home cage containing maternal odor. A clear preference for the area with soiled bedding was evident in *Myt1l*^+/-^ mouse pups and *Myt1l*^+*/*+^ littermate controls (Fig. 4A). Area crossings reflecting locomotor activity did not differ between genotypes (Fig. 4B). This indicates that social odor processing, social motivation, and basic motor functions are intact in *Myt1l*^+/-^ mouse pups. Body weight and temperature did not differ between genotypes during the homing test at PND 10 (Fig. 4C, D).

### Altered emission of isolation-induced ultrasonic vocalizations in the homing test

**Fig. 5:**
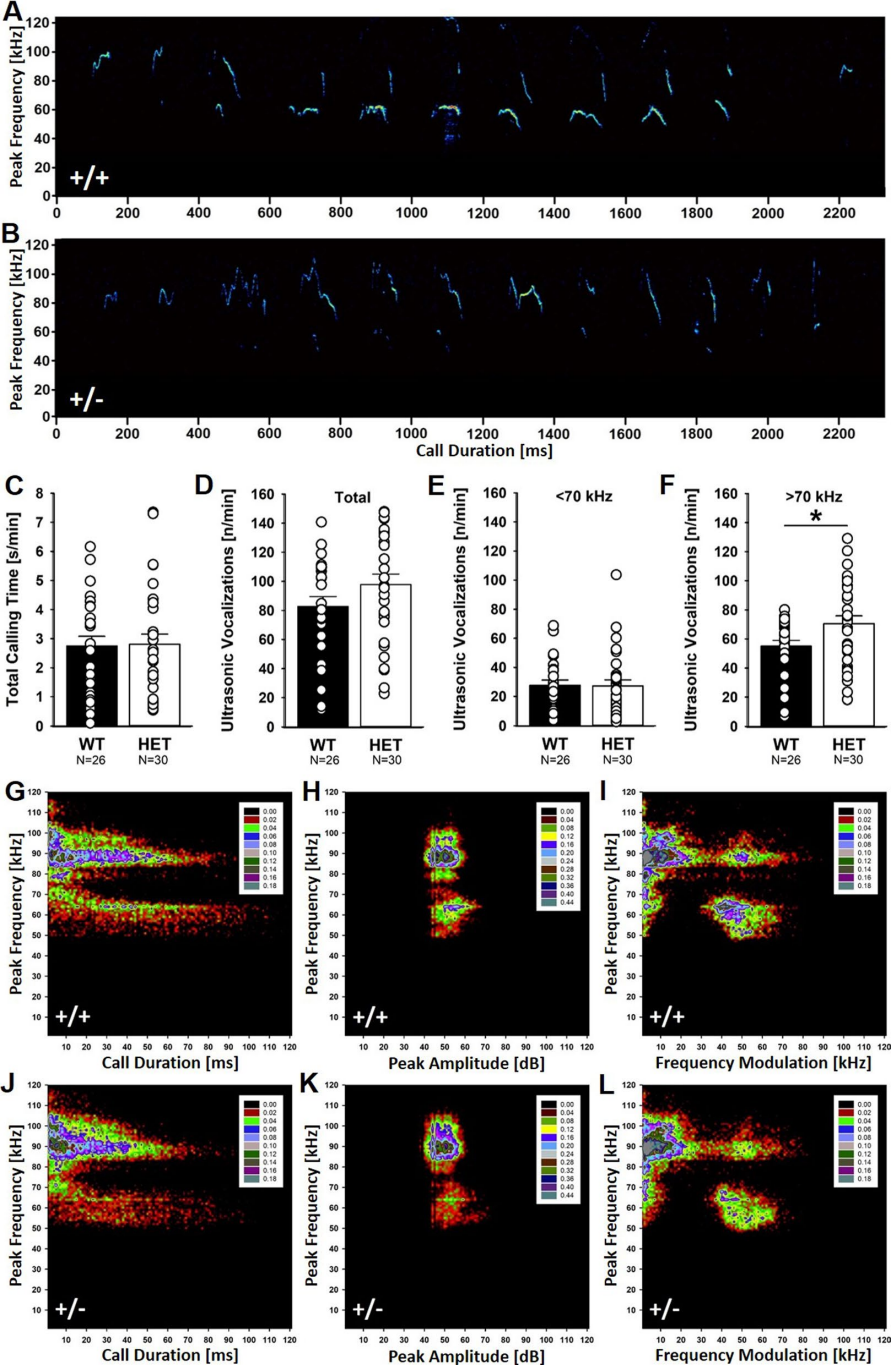
Effects of *Myt1l* haploinsufficiency on the emission of isolation-induced ultrasonic vocalizations in the homing test. **A**, **B** Emission of isolation-induced ultrasonic vocalizations is altered in *Myt1l*^+/-^ mouse pups. Representative spectrograms showing sequences of isolation-induced ultrasonic vocalizations (A: *Myt1l*^+*/*+^ littermate control, B: *Myt1l*^+/-^ mouse pup). Please note the higher peak frequency that is characteristic for many isolation-induced ultrasonic vocalizations emitted by *Myt1l*^+/-^ mouse pups. **C** Total calling time is unchanged in *Myt1l*^+/-^ mouse pups. ANOVA with the between-subject factors genotype (G) and sex (S); all *p* > 0.050. **D** Call number is not altered in *Myt1l*^+/-^ mouse pups. ANOVA with the between-subject factors genotype (G) and sex (S); all *p* > 0.050. **E** Number of calls in the low-frequency cluster is not affected by *Myt1l* haploinsufficiency. ANOVA with the between-subject factors genotype (G) and sex (S); all *p* > 0.050. **F** Number of calls in the high-frequency cluster is increased in *Myt1l*^+/-^ mouse pups. ANOVA with the between-subject factors genotype (G) and sex (S): G: F_1,52_ = 5.760; *p* = .020; all other *p* > 0.050. **G**–**L** Density plots depicting the distribution of individual isolation-induced ultrasonic vocalizations in *Myt1l*^+*/*+^ littermate controls (G-I; ~ 20,000 calls) and *Myt1l*^+/-^ mouse pups (J-L; ~ 30,000 calls). Color coding reflects frequencies as percentages. All data are means ± SEM, combined across males and females; *p < 0.05; **p < 0.01; ***p < 0.001

During the homing test, mouse pups emitted isolation-induced ultrasonic vocalizations (Fig. 5A, B). The latency to start calling, the total time spent calling, and the number of ultrasonic vocalizations emitted were not affected by *Myt1l* haploinsufficiency (Fig. 5C, D). Likewise, acoustic features, such as call duration, peak frequency, peak amplitude, and frequency modulation, were not affected (Additional file 3: Figure S3). Call emission was non-random, and high temporal organization was evident in both genotypes (Additional file 4: Figure S4). However, call clustering was affected by *Myt1l* haploinsufficiency (Fig. 5E, F). Detailed spectrographic analyses of individual ultrasonic vocalizations revealed multiple clusters of call subtypes (Fig. 5G–L). One prominent cluster was characterized by relatively low peak frequencies roughly between 50 and 70 kHz. A second cluster was characterized by relatively high peak frequencies roughly between 80 and 100 kHz. *Myt1l*^+/-^ mouse pups emitted more ultrasonic vocalizations of the high-frequency subtype than *Myt1l*^+*/*+^ littermate controls. The emission of low-frequency ultrasonic vocalizations was not affected by genotype. Alterations in call clustering were not associated with gross developmental measures, and the emission of low-frequency and high-frequency ultrasonic vocalizations was not correlated with body weight and temperature measures obtained during the homing test. Together, this suggests mild alterations in early socio-affective communication in mouse pups before weaning.

### Increased body weight gain but intact body temperature regulation after weaning

**Fig. 6:**
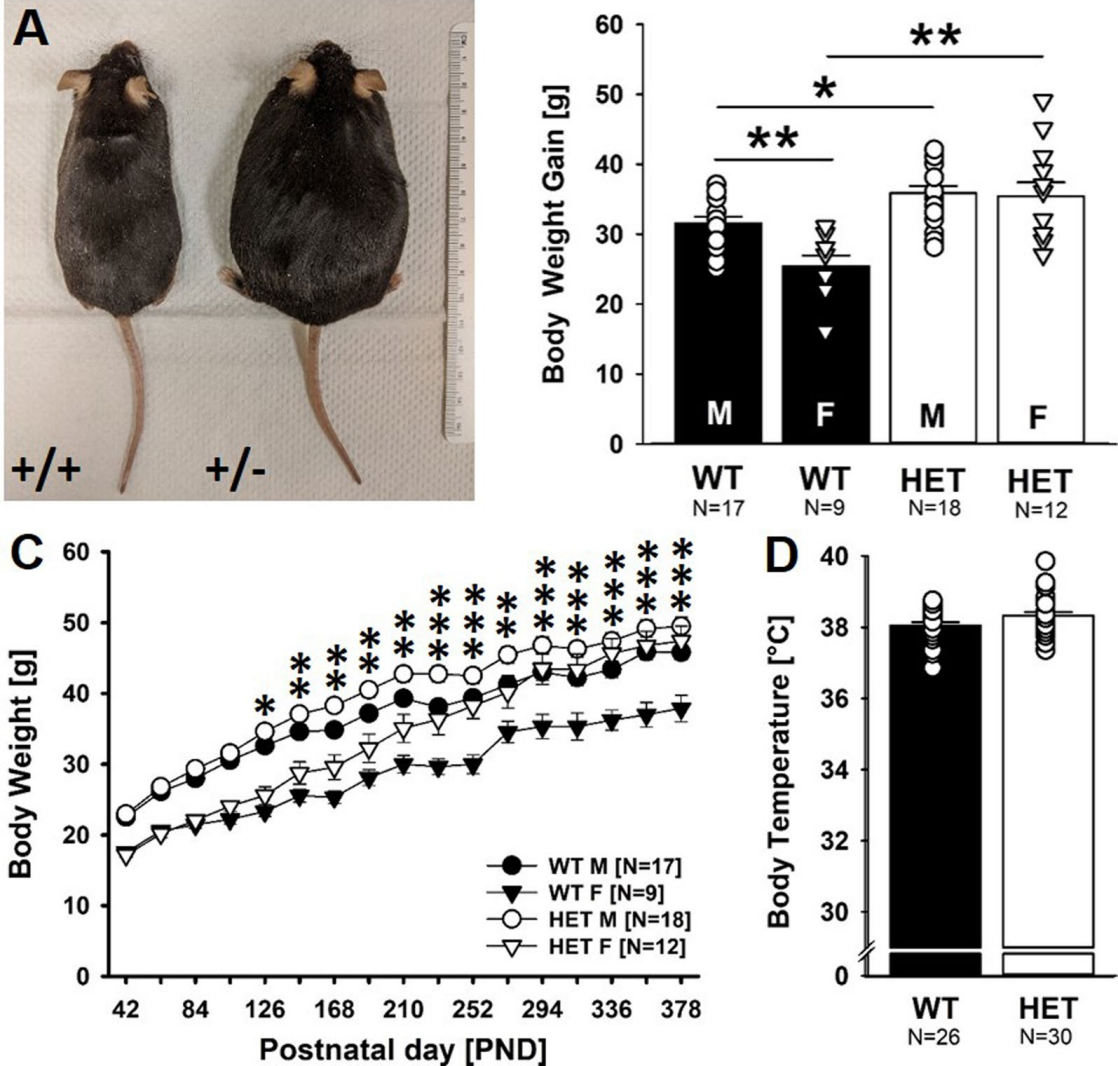
Effects of *Myt1l* haploinsufficiency on body weight gain and body temperature after weaning. **A** Body weight gain is increased in male and female *Myt1l*^+/-^ mice. Representative picture of two females from the same litter (left: *Myt1l*^+*/*+^ littermate control, right: *Myt1l*^+/-^ mouse). **B** Body weight gain after weaning measured at PND 378 (relative to PND 25). ANOVA with the between-subject factors genotype (G) and sex (S), followed by unpaired *t* tests when appropriate. G: F_1,50_ = 26.381; *p* < .001; S: F_1,50_ = 5.532; *p* = .023; GxS: F_1,50_ = 4.054; *p* = .049. **C** Body weight gain trajectories after weaning. Repeated-measures ANOVAs with the between-subject factors genotype (G) and sex (S) and the within-subject factor age (A), followed by individual ANOVAs when appropriate. A: F_16,800_ = 596.784; *p* < .001; AxG: F_16,800_ = 13.965; *p* < .001; AxS: F_16,800_ = 6.314; *p* < .001; AxGxS: F_16,800_ = 3.621; *p* < .001, G: F_1,50_ = 13.428; *p* = .001; S: F_1,50_ = 37.161; *p* < .001; all other *p* > 0.050. **D** Body temperature is not altered in male and female *Myt1l*^+/-^ mice. ANOVA with the between-subject factors genotype (G) and sex (S); all p values > 0.050. Of note, N = 2 male *Myt1l*^+*/*+^ littermate control mice died a natural death between months 11 and 13 without evidence of injuries from fighting and were excluded from the body weight gain and body temperature analyses. All data are means ± SEM, combined across males and females if not otherwise indicated; *p < 0.05; **p < 0.01; ***p < 0.001

As opposed to body weight data during early development, body weight gain was strongly affected by *Myt1l* haploinsufficiency after weaning (Fig. 6A). This effect was sex-dependent (Fig. 6B). In males, *Myt1l*^+/-^ mice gained body weight faster and plateaued at a higher level than *Myt1l*^+*/*+^ littermate controls, yet the overall effect was small (Fig. 6C). In females, however, body weight gain was substantially higher in *Myt1l*^+/-^ mice compared to *Myt1l*^+*/*+^ littermates. In fact, female *Myt1l*^+/-^ mice gained so much body weight during late adulthood that they reached body weight levels of male *Myt1l*^+*/*+^ littermates. In *Myt1l*^+*/*+^ littermates, in contrast, the expected sex difference was present, with males weighing more than females (Fig. 6C). Body temperature at 14 months of age was not affected by *Myt1l* haploinsufficiency, irrespective of sex (Fig. 6D).

### Increased locomotor activity and impaired habituation learning in the activity box

**Fig. 7:**
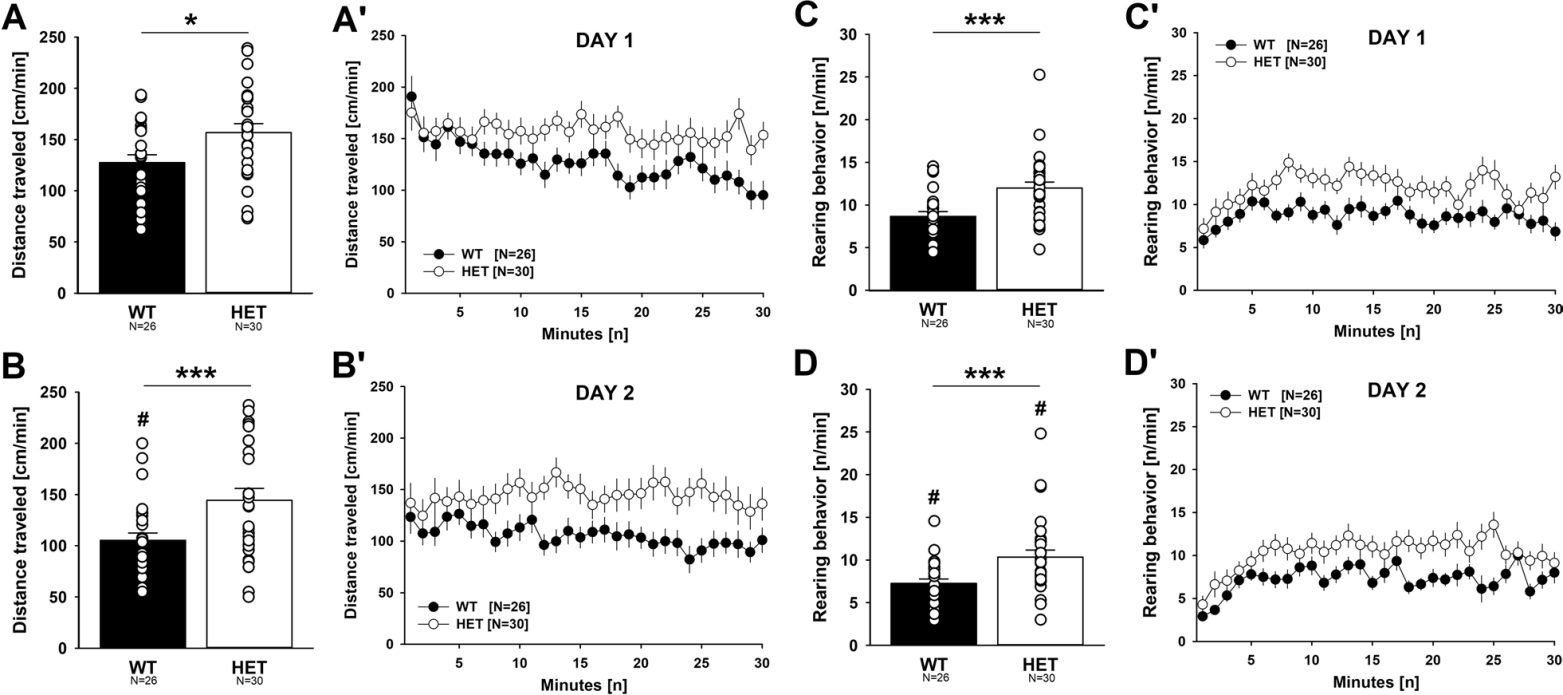
Effects of *Myt1l* haploinsufficiency on locomotor activity and habituation learning in the activity box. **A**–**A′** Locomotor activity is increased in *Myt1l*^+/-^ mice. Repeated-measures ANOVA with the between-subject factors genotype (G) and sex (S) and the within-subject factor time (T). T: F_29,1508_ = 3.848; p < .001; TxG: F_29,1508_ = 1.819; *p* = .005; TxS: F_29,1508_ = 1.634; *p* = .018; G: F_1,52_ = 8.682; *p* = .005; S: F_1,52_ = 11.948; *p* = .001; all other *p* > 0.050. **B–B′** Rearing behavior is increased in *Myt1l*^+/-^ mice. Repeated-measures ANOVAs with the between-subject factors genotype (G) and sex (S) and the within-subject factor time (T). T: F_29,1508_ = 2.760; *p* < .001; G: F_1,52_ = 17.237; *p* < .001; GxS: F_1,52_ = 5.395; *p* = .024; all other *p* > 0.050. **C–C′** Locomotor activity remains high in *Myt1l*^+/-^ mice during the second exposure to the activity box. Repeated-measures ANOVAs with the between-subject factors genotype (G) and sex (S) and the within-subject factor time (T). TxS: F_29,1508_ = 1.853; *p* = .004; G: F_1,52_ = 15.266; *p* < .001; S: F_1,52_ = 22.731; *p* < .001; GxS: F_1,52_ = 9.918; *p* = .003; all other *p* > 0.050. **D–D′** Rearing behavior remains high in *Myt1l*^+/-^ mice during the second exposure to the activity box. Repeated-measures ANOVAs with the between-subject factors genotype (G) and sex (S) and the within-subject factor time (T). T: F_29,1508_ = 4.570; *p* < .001; TxS: F_29,1508_ = 2.086; *p* = .001; G: F_1,52_ = 11.525; *p* < .001; all other *p* > 0.050. All data are means ± SEM, combined across males and females; *p < 0.05; **p < 0.01; ***p < 0.001; ^#^*p* < 0.05 versus day 1

At the behavioral level, *Myt1l* haploinsufficiency led to hyperactivity. During the first exposure to the activity box for 30 min, *Myt1l*^+/-^ mice displayed higher levels of locomotor activity than *Myt1l*^+*/*+^ littermate controls (Fig. 7A). Increased locomotor activity was due to a habituation deficit. While *Myt1l*^+*/*+^ littermates displayed prominent habituation and locomotor activity substantially decreased toward the end of testing, within-session habituation was weak in *Myt1l*^+/-^ mice and locomotor activity remained high. The elevated level of locomotor activity was paralleled by increased rearing behavior (Fig. 7B). *Myt1l*^+/-^ mice displayed more rearing behavior than *Myt1l*^+*/*+^ littermates. During the second exposure to the activity box the next day, locomotor activity levels of *Myt1l*^+/-^ mice remained higher than the ones displayed by *Myt1l*^+*/*+^ littermates (Fig. 7C). While *Myt1l*^+*/*+^ littermates displayed prominent habituation and locomotor activity was substantially lower during the second exposure than during the first exposure, between-session habituation was weak in *Myt1l*^+/-^ mice and locomotor activity remained high. The increase in locomotor activity was again paralleled by elevated levels of rearing behavior (Fig. 7D). Rearing behavior was lower during the second exposure than during the first exposure in both genotypes. Genotype effects were similar in males and females, and sex had only a minor modulatory role.

### Reduced anxiety-related behavior but enhanced nest building activities

**Fig. 8:**
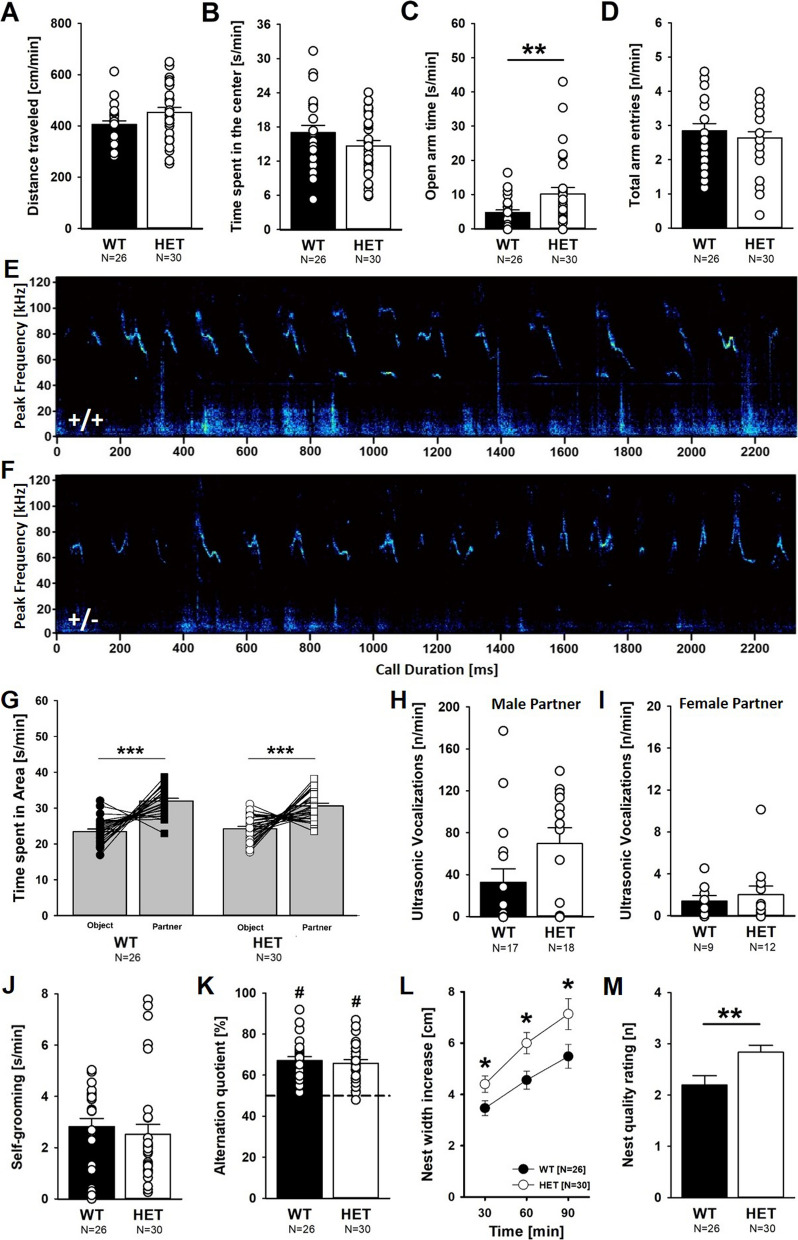
Effects of *Myt1l* haploinsufficiency on anxiety-related behavior, social behavior, interaction-induced ultrasonic vocalizations, self-grooming behavior, spatial working memory, and nest building. **A** Distance traveled in the open field. ANOVA with the between-subject factors genotype (G) and sex (S). S: F_1,52_ = 8.198; *p* = .006; all other *p* > 0.050. **B** Time spent in the center of the open field. ANOVA with the between-subject factors genotype (G) and sex (S). S: F_1,52_ = 18.534; *p* < .001; all other *p* > 0.050. **C** Time spent in the open arms of the elevated plus maze. ANOVA with the between-subject factors genotype (G) and sex (S). G: F_1,52_ = 8.747; *p* = .005; S: F_1,52_ = 7.494; *p* = .008; all other *p* > 0.050. **D** Entries into open and closed arms of the elevated plus maze. ANOVA with the between-subject factors genotype (G) and sex (S). S: F_1,52_ = 5.382; *p* = .024; all other *p* > 0.050. **E**, **F** Emission of female-induced ultrasonic vocalizations is not altered in *Myt1l*^+/-^ mice. Representative spectrograms showing sequences of female-induced ultrasonic vocalizations (A: *Myt1l*^+*/*+^ littermate control, B: *Myt1l*^+/-^ mouse). Please note the presence of low-frequency noise due to locomotor activity. **G** Social approach behavior is intact in *Myt1l*^+/-^ mice. Repeated-measures ANOVA with the between-subject factors genotype (G) and sex (S) and the within-subject factor preference (P). P: F_1,52_ = 45.199; *p* < .001; all other *p* > 0.050. **H** Call number is not altered during male–female social interaction in *Myt1l*^+/-^ mice. ANOVA with the between-subject factor genotype (G); all *p* > 0.050. **I** Call number is not altered during female–female social interaction in *Myt1l*^+/-^ mice. ANOVA with the between-subject factor genotype (G); all *p* > 0.050. **J** Self-grooming. ANOVA with the between-subject factors genotype (G) and sex (S); all *p* > 0.050. **K** Alternation quotient Y-maze. ANOVA with the between-subject factors genotype (G) and sex (S); all *p* > 0.050. **L** Nest width. Repeated-measures ANOVA with the between-subject factors genotype (G) and sex (S) and the within-subject factor time (T), followed by unpaired *t* tests when appropriate. T: F_1,260_ = 25.511; *p* < .001; G: F_1,52_ = 5.591; *p* = .022; all other *p* > 0.050. **M** Nest quality. ANOVA with the between-subject factors genotype (G) and sex (S). G: F_1,52_ = 9.644; *p* = .003; all other *p* > 0.050. All data are means ± SEM, combined across males and females if not otherwise indicated; *p < 0.05; **p < 0.01; ***p < 0.001. ^#^*p* < 0.05 versus 50% chance level

We next assessed anxiety-related behavior. When tested in a large open field under aversive conditions with bright light for 10 min, *Myt1l* haploinsufficiency did not exert prominent effects on locomotor activity (Fig. 8A) and the time spent in the center did not differ between *Myt1l*^+/-^ mice and *Myt1l*^+*/*+^ littermate controls (Fig. 8B). The number of fecal boli did not differ between genotypes. In the elevated plus maze, however, *Myt1l* haploinsufficiency led to reduced anxiety-related behavior and *Myt1l*^+/-^ mice spent more time in the open arms than *Myt1l*^+*/*+^ littermates (Fig. 8C). The number of open and closed arm entries did not differ between genotypes, suggesting that the increased time spent in open arms is not a simple reflection of hyperactivity (Fig. 8D). Social interaction behavior in adulthood appeared not to be affected by *Myt1l* haploinsufficiency. The emission of ultrasonic vocalizations during social interactions was intact (Fig. 8E, F). During male–female social interactions, emission of ultrasonic vocalizations was high in pairs irrespective of the genotype of the male mouse (Fig. 8H). Relatively low levels of ultrasonic vocalizations were seen during female–female social interactions, and there was no evidence for genotype differences (Fig. 8I). In fact, social approach behavior in the three-chamber assay was seen in *Myt1l*^+/-^ mice and *Myt1l*^+*/*+^ littermates (Fig. 8G).

When assessing repetitive behavior during exposure to a novel cage with corn cob bedding, self-grooming did not differ between genotypes (Fig. 8J). Likewise, spatial working memory in the Y-maze was not affected. *Myt1l*^+/-^ mice performed similarly to *Myt1l*^+*/*+^ littermate controls, and the alternation quotient did not differ between genotypes (Fig. 8K). Both genotypes displayed the expected preference for sequentially visiting each of the three arms over visiting one arm twice in three consecutive entries, reflecting intact spatial working memory. Nest building behavior, in contrast, was affected by *Myt1l* haploinsufficiency (Fig. 8L). When exposed to a square of pressed cotton, *Myt1l*^+/-^ mice used the cotton to build wider nests than *Myt1l*^+*/*+^ littermates. Nest width increased faster and to higher levels in *Myt1l*^+/-^ mice as compared to *Myt1l*^+*/*+^ littermates. This led to higher nest quality ratings in *Myt1l*^+/-^ mice (Fig. 8M). Of note, nest height was not affected by genotype.

### Reduced motor performance but intact motor learning on the accelerated rotarod

**Fig. 9:**
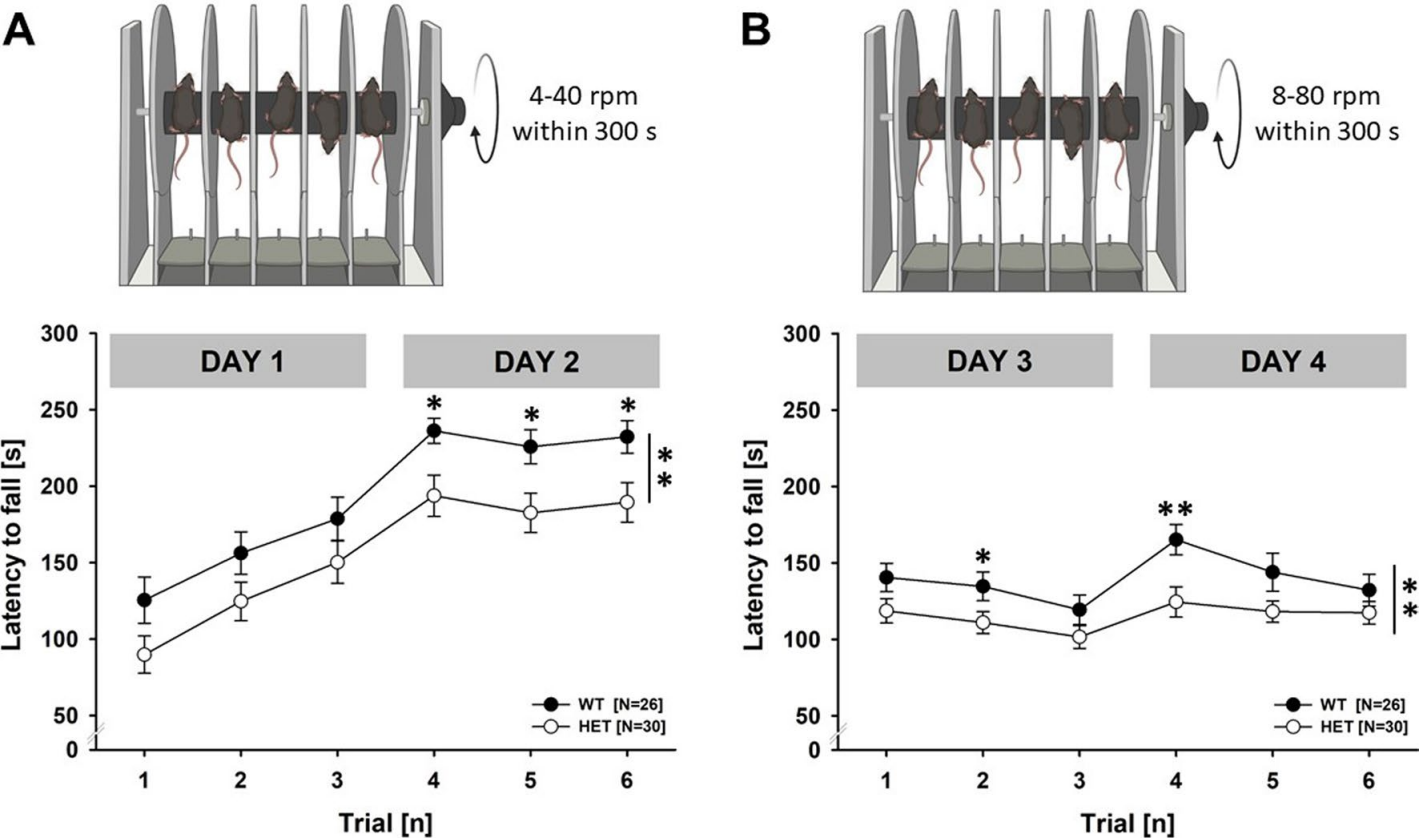
Effects of *Myt1l* haploinsufficiency on motor performance and learning on the accelerating rotarod. **A** Motor performance in the standard task with an accelerating rotarod (4–40 rpm) is reduced in *Myt1l*^+/-^ mice and reflected in a lower latency to fall, despite evidence for intact motor learning. Repeated-measures ANOVA with the between-subject factors genotype (G) and sex (S) and the within-subject factor trial (T), followed by unpaired *t* tests when appropriate. T: F_1,260_ = 51.953; *p* < .001; G: F_1,52_ = 7.309; *p* = .009; S: F_1,52_ = 11.197; *p* = .002; all other *p* > 0.050. **B** Motor performance in the challenge task with an accelerating rotarod (8–80 rpm) is reduced in *Myt1l*^+/-^ mice and reflected in a lower latency to fall. Repeated-measures ANOVA with the between-subject factors genotype (G) and sex (S) and the within-subject factor trial (T), followed by unpaired *t* tests when appropriate. T: F_1,260_ = 6.701; *p* < .001; TxS: F_5,260_ = 2.512; *p* = .030; G: F_1,52_ = 8.933; *p* = .004; S: F_1,52_ = 21.467; *p* < .001; all other *p* > 0.050. All data are means ± SEM, combined across males and females; *p < 0.05; **p < 0.01; ***p < 0.001

Two versions of the rotarod task were used. First, we applied the standard task with an accelerating rod from 4 to 40 rpm within 300 s (Fig. 9A). *Myt1l* haploinsufficiency had a prominent effect on motor performance. Although motor performance gradually improved across trials in both genotypes on the first day of training in line with intact motor learning, overall motor performance was lower in *Myt1l* ^+/-^mice than *Myt1l*^+*/*+^ littermate controls. The genotype difference was particularly prominent on the second day, where the latency to fall was lower in *Myt1l*^+/-^ mice than *Myt1l*^+*/*+^ littermates during all three trials. Importantly, genotypes did not differ in the latency to make one complete revolution while hanging on the rotarod, meaning that *Myt1l*^+*/*+^ littermates were able to remain on the rotarod for longer periods of time under challenging conditions than *Myt1l*^+/-^ mice. This is also reflected in a higher number of turns before falling in *Myt1l*^+*/*+^ littermates than *Myt1l*^+/-^ mice. To test this further, we next expanded the standard range of acceleration from 4–40 rpm to 8–80 rpm within 300 s (Fig. 9B). As expected, the latency to fall was clearly lower in the challenge task compared to the standard task. Similar to the standard task, however, motor performance was lower in *Myt1l*^+/-^ mice than *Myt1l*^+*/*+^ littermates, as reflected in a lower latency to fall. The latency to make one complete revolution did not differ between genotypes. Again, *Myt1l*^+*/*+^ littermates made a higher number of turns before falling than *Myt1l*^+/-^ mice. Together, this suggests that reduced motor performance in *Myt1l*^+/-^ mice occurs irrespective of task difficulty, both in the standard task and in the challenge task. The effect appears be driven by a higher motivation and/or capacity of *Myt1l*^+*/*+^ littermates to remain on the accelerating rotarod after a complete revolution.

### Intact spatial learning and reversal learning

**Fig. 10:**
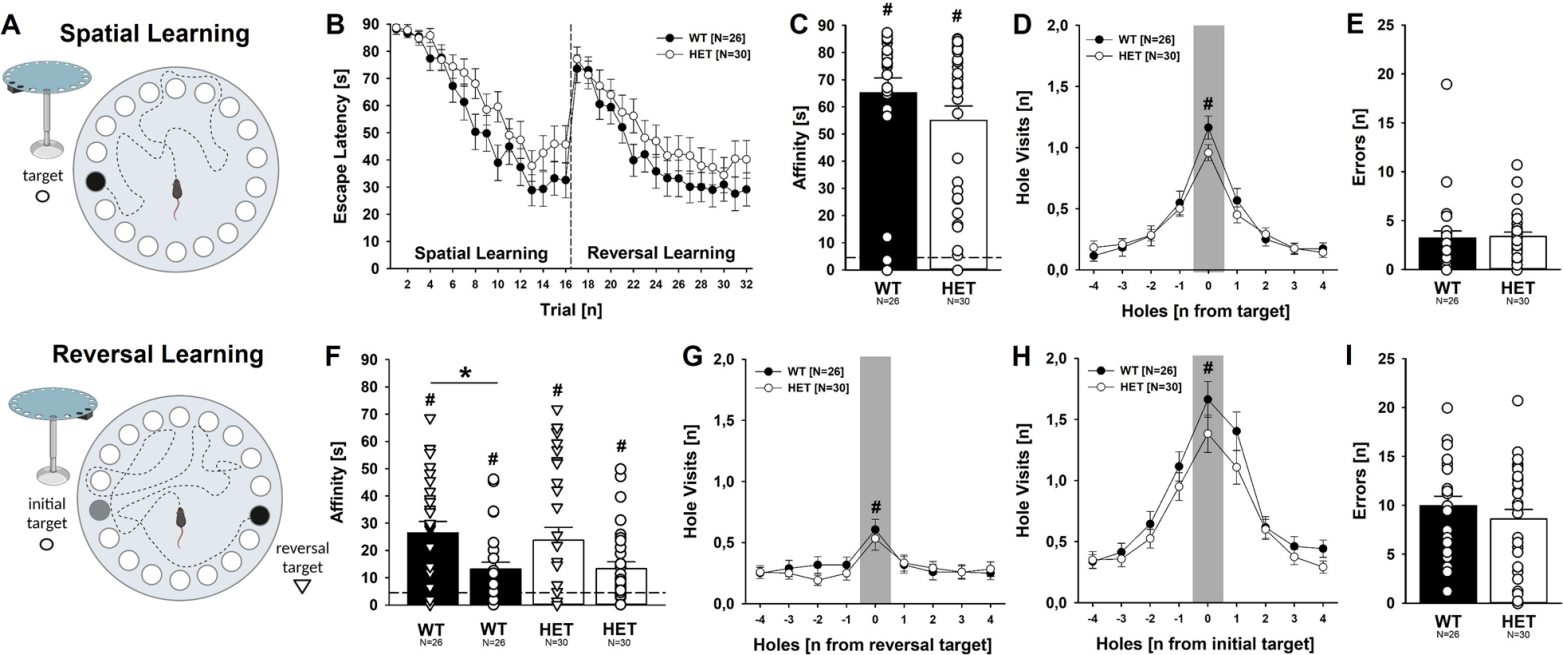
Effects of *Myt1l* haploinsufficiency on spatial learning and reversal learning. **A** Spatial learning and reversal learning in the Barnes maze is intact in *Myt1l*^+/-^ mice. **B** Intact spatial learning and reversal learning is reflected in a decrease in the latency needed to reach the target hole and the reversal target hole, respectively. Repeated-measures ANOVA with the between-subject factors genotype (G) and sex (S) and the within-subject factor trial (T). For spatial learning, T: F_15,780_ = 41.186; *p* < .001; S: F_1,52_ = 6.936; *p* = .011; all other *p* > 0.050. For reversal learning, T: F_15,780_ = 28.583; *p* < .001; S: F_1,52_ = 4.138; *p* = .047; all other *p* > 0.050. **C** Affinity for the target hole during spatial learning is unchanged in *Myt1l*^+/-^ mice. ANOVA with the between-subject factors genotype (G) and sex (S); all *p* > 0.050. **D** Intact spatial learning in *Myt1l*^+/-^ mice is also reflected in a clear preference for visiting the target hole during the last spatial learning day. Gray highlighting indicates target. Repeated-measures ANOVA with the between-subject factors genotype (G) and sex (S) and the within-subject factor preference (P). P: F_8,416_ = 70.310; *p* < .001; all other *p* > 0.050. **E** Primary errors during spatial learning are unchanged in *Myt1l*^+/-^ mice. ANOVA with the between-subject factors genotype (G) and sex (S); all *p* > 0.050. **F** Affinity for the reversal target hole is slightly reduced in *Myt1l*^+/-^ mice. ANOVA with the between-subject factors genotype (G) and sex (S); all *p* > 0.050. Paired *t* test for comparing reversal target hole (triangle) and initial target hole (circle); *Myt1l*^+/-^ mice: t_29_ = 1.615; *p* = .117; *Myt1l*^+*/*+^ mice: t_25_ = 2.222; *p* = .036. **G**, **H** Despite a clear preference for the initial target hole, intact reversal learning in *Myt1l*^+/-^ mice is also reflected in the rapid development of a secondary preference for visiting the reversal target hole during the first reversal learning day. Gray highlighting indicates target. Repeated-measures ANOVA with the between-subject factors genotype (G) and sex (S) and the within-subject factor preference (P). For the initial target hole, P: F_8,416_ = 72.597; *p* < .001; all other *p* > 0.050. For the reversal target hole, P: F_8,416_ = 9.625; *p* < .001; PxS: F_8,416_ = 3.657; *p* < .001; all other *p* > 0.050. **I** Primary errors during reversal learning are unchanged in *Myt1l*^+/-^ mice. ANOVA with the between-subject factors genotype (G) and sex (S); all *p* > 0.050. All data are means ± SEM, combined across males and females; *p < 0.05; **p < 0.01; ***p < 0.001. ^#^*p* < 0.05 versus 4.5s chance level or adjacent holes to target

We next studied spatial learning and reversal learning. First, we examined the effects of *Myt1l* haploinsufficiency on spatial learning abilities in the Barnes maze under direct bright white light conditions (Fig. 10A, upper part). As expected, the mice naturally sought out the target escape hole, driven by their desire to escape brightly lit and exposed environments. During the consecutive four-day training period, the mice learned the spatial location of the target hole and the latency needed to reach the target hole decreased across trials (Fig. 10B, left). The decrease in latency displayed by *Myt1l*^+/-^ mice was slightly weaker but overall similar to *Myt1l*^+*/*+^ littermate controls, indicating an intact ability to spatially navigate the maze to find the target escape hole. Moreover, a clear preference for the target hole was evident during the last spatial learning day. This was reflected in the time spent inside or in close proximity to the target hole. In fact, affinity for the target hole was high irrespective of genotype, albeit slightly lower in *Myt1l*^+/-^ mice than in *Myt1l*^+/+^ littermates (Fig. 10C). A clear preference for the target hole was also reflected in the pattern of hole visits. The target hole was visited clearly more often than adjacent holes, reflecting intact spatial memory with high resolution. The preference for the target hole was particularly strong in *Myt1l*^+*/*+^ littermates but weaker in *Myt1l*^+/-^ mice (Fig. 10D). Primary errors did not differ between genotypes (Fig. 10E).

Next, we tested reversal learning abilities by rotating the target hole by ~ 180° (Fig. 10A, lower part). The mice were again trained for four consecutive days. As expected, the latency needed to reach the reversal target hole was high at the beginning, but decreased across trials (Fig. 10B, right). The decrease in latency displayed by *Myt1l*^+/-^ mice was again slightly weaker but overall similar to *Myt1l*^+*/*+^ littermates, indicating intact reversal learning abilities. Moreover, a preference for the reversal target hole emerged already during the first reversal learning day and affinity for the reversal target hole was evident irrespective of genotype, albeit the level of affinity was slightly lower in *Myt1l*^+/-^ mice than in *Myt1l*^+*/*+^ littermates. When compared to the initial target hole, the affinity for the reversal target hole was higher in *Myt1l*^+*/*+^ littermates but did not reach statistical significance in *Myt1l*^+/-^ mice (Fig. 10F). While affinity for the reversal target hole indicates intact reversal learning abilities, the pattern of hole visits reflects strong competing memory traces associated with the location of the initial target hole. In fact, the pattern of hole visits indicates that a clear preference for the initial target hole was still evident. Although a secondary preference for the reversal target hole rapidly developed (Fig. 10G), the initial target hole was visited clearly more often than the reversal target hole and the difference between initial target hole and adjacent holes was a lot more prominent than the difference between reversal target hole and adjacent holes (Fig. 10H). Importantly, these preferences occurred irrespective of genotype. Primary errors did not differ between genotypes (Fig. 10I). Together, this indicates that spatial learning and reversal learning are intact in *Myt1l*^+/-^ mice, despite minor evidence for worse performance.

### Enhanced acoustic startle reactivity but intact sensorimotor gating

**Fig. 11:**
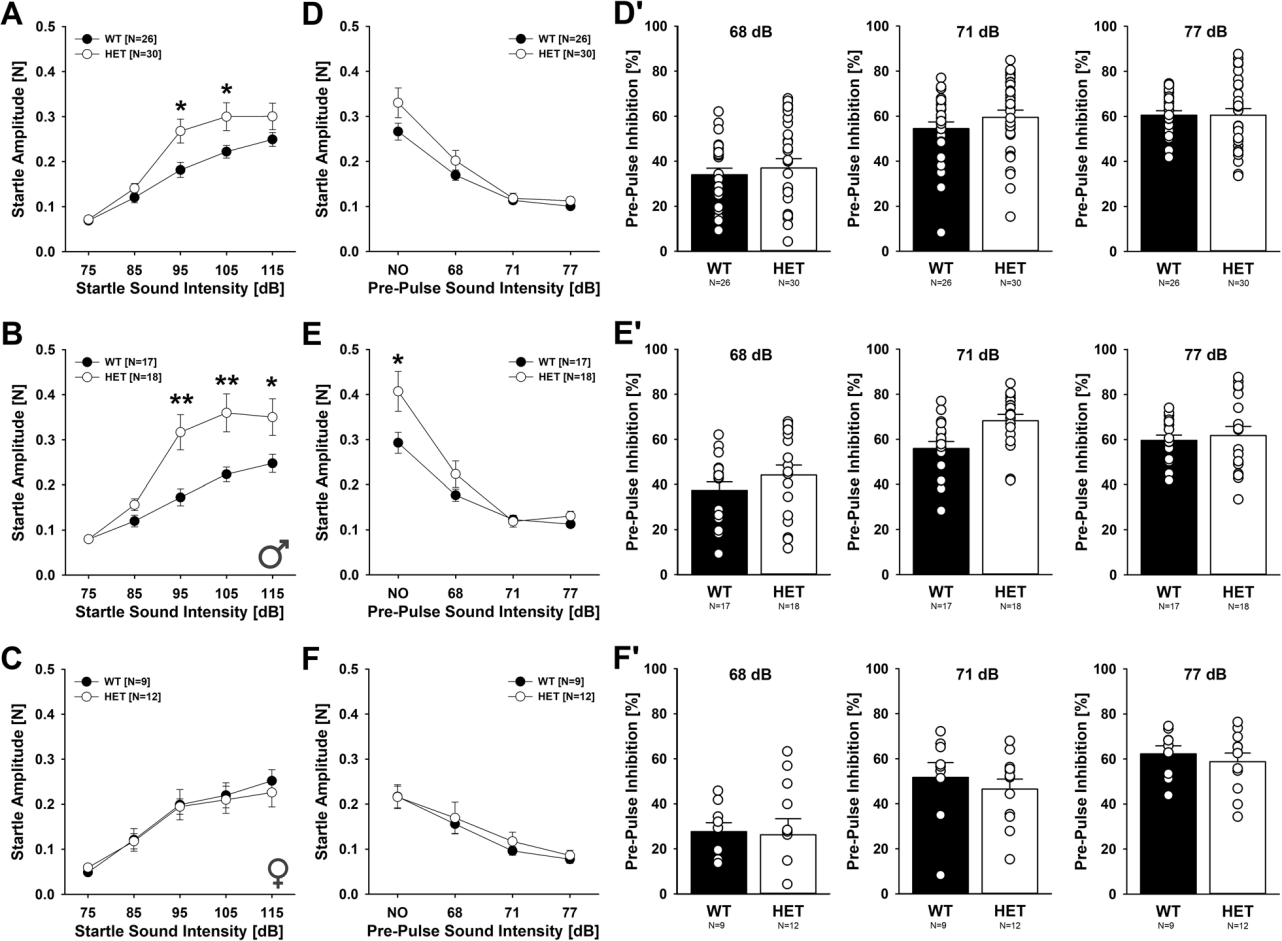
Effects of *Myt1l* haploinsufficiency on acoustic startle reactivity and pre-pulse inhibition of acoustic startle. **A**–**C** Acoustic startle reactivity is increased in male but not female *Myt1l*^+/-^ mice. Repeated-measures ANOVAs with the between-subject factors genotype (G) and sex (S) and the within-subject factor startle sound intensity (I), followed by unpaired *t* tests when appropriate. I: F_4,208_ = 84.359; p < .001; IxGxS: F_4,208_ = 3.648; *p* = .007, S: F_1,52_ = 4.662; *p* = .035; GxS: F_1,52_ = 4.574; *p* = .037; all other *p* > 0.050. **D**–**F** Pre-pulse inhibition of acoustic startle is unchanged in male and female *Myt1l*^+/-^ mice. Repeated-measures ANOVAs with the between-subject factors genotype (G) and sex (S) and the within-subject factor pre-pulse sound intensity (I), followed by unpaired *t* tests when appropriate. I: F_3,156_ = 95.448; *p* < .001; IxS: F_3,156_ = 9.664; *p* < .001; IxGxS: F_3,156_ = 3.071; *p* = .030; S: F_1,52_ = 8.443; *p* = .005; all other *p* > 0.050. **D′–F′** Pre-pulse inhibition of acoustic startle is unchanged in male and female *Myt1l*^+/-^ mice also after correcting for baseline differences in startle reactivity. Repeated-measures ANOVAs with the between-subject factors genotype (G) and sex (S) and the within-subject factor pre-pulse sound intensity (I), followed by unpaired *t* tests when appropriate. I: F_2,104_ = 80.919; *p* < .001; IxS: F_2,104_ = 5.880; *p* = .004; S: F_1,52_ = 6.643; *p* = .013; all other *p* > 0.050. All data are means ± SEM, combined across males and females if not otherwise indicated; *p < 0.05; **p < 0.01; ***p < 0.001

*Myt1l* haploinsufficiency led to increased acoustic startle responses (Fig. 11A). This effect was sex-dependent. In males, prominent genotype differences were seen and startle reactivity was potentiated in *Myt1l*^+/-^ mice during the exposure to pulses with moderate and high sound intensities of 95, 105, and 115 dB, possibly reflecting higher sensitivity and/or stronger fear response to loud sound. No differences were seen in response to low sound intensities of 75 and 85 dB (Fig. 11B). In females, *Myt1l*^+/-^ mice displayed acoustic startle responses similar to *Myt1l*^+*/*+^ littermate controls (Fig. 11C). Effects of *Myt1l* haploinsufficiency were also evident during assessment of pre-pulse inhibition of acoustic startle (Fig. 11D, D′). Replicating genotype differences in startle reactivity, males but not females displayed a potentiated startle response during presentation of a pulse of 115 dB in the absence of a pre-pulse. However, pre-pulse inhibition of acoustic startle was not affected by *Myt1l* haploinsufficiency irrespective of sex, albeit pre-pulse inhibition tended to be slightly stronger in male *Myt1l*^+/-^ mice than *Myt1l*^+*/*+^ littermates after correcting for baseline differences in startle reactivity (Fig. 11E, E′). No such effect was seen in females (Fig. 11F, F′). This shows that sensorimotor gating is intact in *Myt1l*^+/-^ mice.

### Reduced fear acquisition and contextual fear memory retrieval

**Fig. 12:**
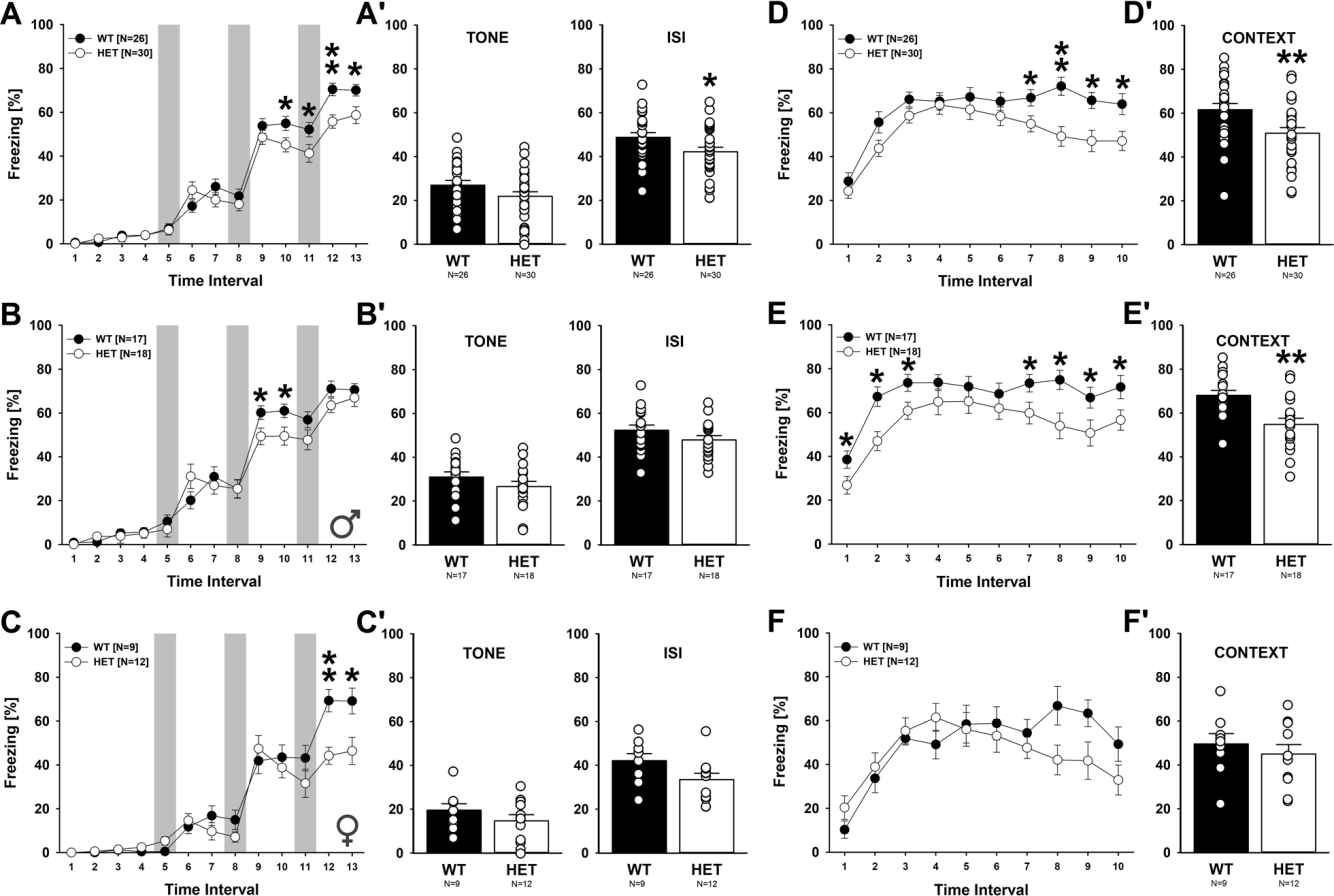
Effects of *Myt1l* haploinsufficiency on fear conditioning. **A**–**C** Fear-related freezing behavior is enhanced in male and female *Myt1l*^+/-^ mice during acquisition on the training day. Gray highlighting indicates tone presentations (1 min inter-stimulus intervals). Repeated-measures ANOVAs with the between-subject factors genotype (G) and sex (S) and the within-subject factor time (T), followed by unpaired *t* tests when appropriate. T: F_12,624_ = 190.370; *p* < .001; TxG: F_12,624_ = 3.535; *p* < .001; TxS: F_12,624_ = 2.355; *p* = .006; TxGxS: F_12,624_ = 2.015; *p* = .021; G: F_1,52_ = 4.803; *p* = .033; S: F_1,52_ = 25.623; *p* < .001; all other *p* > 0.050. **D**–**F** Fear-related freezing behavior is enhanced in male but not female *Myt1l*^+/-^ mice during contextual recall. Repeated-measures ANOVAs with the between-subject factors genotype (G) and sex (S) and the within-subject factor time (T), followed by unpaired *t* tests when appropriate. T: F_9,468_ = 23.286; *p* < .001; TxG: F_9,468_ = 2.660; *p* = .005; G: F_1,52_ = 6.682; *p* = .013; S: F_1,52_ = 16.678; *p* < .001; all other *p* > 0.050. All data are means ± SEM, combined across males and females if not otherwise indicated; *p < 0.05; **p < 0.01; ***p < 0.001

Finally, we applied a fear conditioning paradigm and quantified fear-related freezing behavior during acquisition, cued recall, and contextual recall. On the training day, freezing behavior increased with tone-shock pairings during acquisition, as expected. This increase in freezing behavior was affected by *Myt1l* haploinsufficiency. Genotype differences emerged with repeated tone-shock pairings and were most prominent toward the end of acquisition (Fig. 12A). In both sexes, *Myt1l*^+/-^ mice displayed less freezing behavior than *Myt1l*^+*/*+^ littermate controls (Fig. 12B, C). While no prominent differences were seen during tone presentations,  relatively low levels of freezing behavior were seen in *Myt1l*^+/-^ mice between tone presentations (Fig. 12A′), irrespective of sex (Fig. 12B′, C′). During contextual recall 24 h after training, freezing behavior was likewise affected by *Myt1l* haploinsufficiency. When placed back into the original conditioning chamber, *Myt1l*^+/-^ mice displayed less freezing behavior than *Myt1l*^+*/*+^ littermates (Fig. 12D, D′). This effect was particularly prominent in males (Fig. 12E, E′), while not as clear in females, possibly because of lower freezing levels in females than in males (Fig. 12F, F′). In contrast to contextual recall, cued recall was not affected by *Myt1l* haploinsufficiency. Irrespective of sex, *Myt1l*^+/-^ mice did not differ from *Myt1l*^+*/*+^ littermates during tone presentation (Additional file 5: Figure S5). This suggests that *Myt1l* haploinsufficiency has a negative impact on contextual fear memory retrieval, while cued fear memory retrieval appears to be intact. However, it has to be noted that surprisingly high levels of freezing were evident in the altered context, without the typical sharp increase in freezing observed at the onset of the cue. This seems to indicate a robust generalization of the fear response to the new context, rather than specific cue-dependent fear learning. Nevertheless, *Myt1l*^+/-^ mice did not differ from *Myt1l*^+*/*+^ littermates during exposure to the altered context used for cued recall, showing that the genotype differences are specifically seen during contextual recall but are not due to nonspecific differences in activity levels.

## Discussion

To investigate the consequences of the genetic deletion of *Myt1l* in mice, we generated a Myt1l-deficient mouse line using CRISPR/Cas9 gene editing. Our Myt1l-deficient mouse line differs in important aspects from another recently described mouse model for *Myt1l* haploinsufficiency that was generated independently from ours by Chen et al. [38]. First, the kind of genetic mutation introduced differs substantially. While we produced a frameshift mutation in the first coding exon predicting a truncated protein of just 77 amino acids, Chen et al. inserted a G at position c.2130 of the mouse coding sequence, corresponding to a c.3005 mutation in the human gene found in a patient. The Chen et al. mutation results in a stop codon at c.2142 resulting in a predicted truncated protein of 713 amino acids. The predicted molecular weight of these 713 amino acids is 80.6 kDa, and Chen et al. correctly report that no band is visible in this size range in their mutant mice. Given that the 1187 amino acid long full-length protein is predicted to be only 132.9 kDa, it runs, however, at around ~ 170 kDa, it is very possible that the 713 amino acid fragment has a molecular weight over 100 kDa. Indeed, upon closer inspection of the Western blot published by Chen et al. (their Suppl. Fig. S1I), there could be an additional band in the mutant brains just above 100kD, yet several unspecific bands in that blot complicate the interpretation. Importantly, in our allele we certainly do find an unexpected additional band by Western analysis of a size of about 158 kDa. We confirmed this extra band with another independent antibody. At position 99, there is another Methionine which could serve as alternative start codon in our mutant allele, which would result in a N-terminally truncated Myt1l protein of about 10% shorter sequence and thus compatible with the ~ 13% observed decreased size.

With regards to phenotypic characterization, the two studies are complementary but some assays were similar. Most notably, we demonstrate that the neuron-specific transcription factor Myt1l is critical for survival beyond the first day of life in mice. This finding was also reported by Chen et al. despite the different *Myt1l* mutation [38]. However, our allele did not produce any substantial neurogenesis defects as determined by quantitative image analysis of Sox2 + VZ progenitors, Tbr2 + basal progenitors, and Ctip2 + neurons. In contrast, Chen et al. reported a decrease in Sox2 + cells with normal numbers of Tbr2 + cells and Tbr1 + neurons. They also reported a decreased EdU incorporation consistent with reduced Sox2 staining, which is very interesting because Myt1l is not expressed in these proliferative cells. The overall histological brain organization of our *Myt1l* mutant mice was normal. Chen et al. do not report detailed histological analyses but report decreased volumes of the corpus callosum and cortex based on magnetic resonance imaging-based fractional anisotropy and diffusion tensor imaging. The smaller brain structures produced by the Chen et al. allele may be well explained by the observed decreased progenitor cell proliferation. In our mice, however, we do not find any neurogenesis defects; hence, Myt1l’s requirement for organismal postnatal survival must be related to Myt1l’s function in postmitotic neurons where it is physiologically expressed.

By means of our newly generated Myt1l-deficient mouse line, we further provided deeper insights into the role of Myt1l during postnatal development and showed that *Myt1l* haploinsufficiency led to obesity and multifaceted behavioral alterations in mice. Specifically, *Myt1l* haploinsufficiency caused a prominent increase in body weight gain after weaning and first reliable differences in body weight were evident at around 4 months of age. While body weight differences were small in male mice, body weight gain was substantial in females, in line with the findings recently obtained in the other mouse model for *Myt1l* haploinsufficiency by Chen et al. [38]. By about 8 months of age, female *Myt1l* heterozygous mice gained so much weight that they reached weight levels typically seen in males. Apparently, the difference in body weight gain was not associated with alterations in basic body functions, such as temperature regulation, as body temperature was not affected by *Myt1l* haploinsufficiency. Fecal boli quantified following open field testing did not differ between genotypes. Obesity is consistent with clinical phenotypes in humans [1–4, 11–15, 17–19, 22]. A recent overview on over 50 known individuals carrying *MYT1L* mutations indicates that about 70% of them were overweight or obese [4]. Obesity is probably driven by polyphagia [1], with reports on increased aggressiveness when food is denied [14]. In a zebrafish model, knockdown of the *MYT1L* orthologues resulted in altered development of the hypothalamus, most notably a loss of oxytocin expression in the preoptic neuroendocrine area [1]. Given that the preoptic neuroendocrine area was reported to be functional equivalent to the paraventricular nucleus in mammals and lesions of it were shown to cause hyperphagic obesity [52], this might provide a potential mechanism underlying obesity. In future studies, it would thus be interesting to apply metabolic cages and to assess drinking and feeding behavior, circadian activity patterns, and calorimetric measures, such as O_2_ consumption and CO_2_ production, in *Myt1l* heterozygous mice.

First behavioral effects of *Myt1l* haploinsufficiency were seen during early development. When separated from mother and nest, mouse pups emit isolation-induced ultrasonic vocalizations [53]. Such isolation-induced ultrasonic vocalizations serve important communicative functions [54] and induce maternal caregiving behavior [55]. As the first measure of communication in mice, they are commonly assessed in mouse models for ASD and alterations in call emission, acoustic features, and call clustering were repeatedly obtained in relevant model system [56]. In fact, isolation-induced ultrasonic vocalizations were also assessed in the mouse model for *Myt1l* haploinsufficiency by Chen et al. and evidence for enhanced call emission was obtained [38]. While call emission rates and basic acoustic features were not affected in our mouse model for *Myt1l* haploinsufficiency, detailed spectrographic analyses not performed in the other model revealed prominent effects on call clustering. In mouse pups, typically two main clusters of call subtypes can easily be distinguished, i.e., a first cluster characterized by relatively low peak frequencies between 50 and 70 kHz and a second cluster characterized by relatively high peak frequencies between 80 and 100 kHz [57]. Remarkably, *Myt1l* heterozygous mouse pups emitted more high-frequency ultrasonic vocalizations in response to separation from mother and nest. Such changes in call clustering were not associated with gross developmental measures, and the emission of high-frequency ultrasonic vocalizations was not correlated with body weight and temperature. Alterations in call clustering are paralleled by changes in acoustic features known to be important for categorical perception of ultrasonic vocalizations and are thus predicted to alter their communicative function and, consequently, the interaction between mother and pup [55]. Previous studies showed that call clustering is altered in multiple models with relevance to neurodevelopmental disorders, such as ASD [58–60]. Importantly, altered emission of isolation-induced ultrasonic vocalizations was detected despite intact maternal odor preference in the homing test. A clear preference for the area with soiled bedding from the home cage containing maternal odor was evident, and area crossings reflecting locomotor activity did not differ between genotypes. This indicates that social odor processing, social motivation, and basic motor functions during early postnatal development are not affected by *Myt1l* haploinsufficiency. Therefore, alterations in call clustering do not appear to be driven by nonspecific effects. Together, this suggests that *Myt1l* haploinsufficiency causes mild alterations in early socio-affective communication in mouse pups before weaning. In humans, *MYT1L* mutations were strongly associated with speech delay and language impairments, such as echolalia and palilalia [1–4, 11–14, 16–18, 20–22].

While there was no evidence for changes in locomotor activity during early development, *Myt1l* haploinsufficiency led to hyperactivity in adulthood. This is in line with the recent observations made by Chen et al. [38]. Our findings now indicate that the increased locomotor activity was due to impaired habituation learning, often called the simplest form of learning [61]. In fact, both within-session and between-session habituation were weak in *Myt1l* heterozygous mice and locomotor activity remained high throughout testing in an activity box. Elevated locomotor activity was paralleled by increased rearing behavior. The view that increased locomotor activity is driven by impaired habituation learning is supported by the observation that locomotor activity was found to be increased when assessed for 30 min in the activity box but not when tested in an open field for 10 min or other behavioral assays, such as homing test and elevated plus maze. Of note, hyperactivity was seen before obesity emerged and it is unclear whether locomotor activity remained high later during development despite obesity. In humans, hyperactivity is commonly seen in individuals carrying *MYT1L* mutations, but to our knowledge habituation learning was not assessed [1–4, 11–13, 17, 18, 20, 22]. However, ASD and ADHD, both frequently diagnosed in individuals carrying *MYT1L* mutations, were linked to impaired habituation learning [62], suggesting that hyperactivity could be driven by impaired habituation learning in humans as well.

While locomotor activity was enhanced, motor performance on the accelerated rotarod was reduced in *Myt1l* heterozygous mice despite intact motor learning. In the standard task with an accelerating rod from 4 to 40 rpm, motor performance gradually improved across trials in line with intact motor learning, yet overall motor performance was reduced and the latency to fall was low in *Myt1l* heterozygous mice. Reduced motor performance on the accelerated rotarod could be driven by a lower motivation and/or capacity to remain on the rotating accelerating rod and might be associated with emerging obesity and/or muscular hypotonia. This is suggested by the fact that genotypes did not differ in the latency to make one complete revolution while hanging on the rotarod but that *Myt1l* heterozygous mice were less able to remain on the rotarod under challenging conditions, as reflected in less time on the rotating accelerating rod after a complete revolution and a lower number of turns before falling. Consistent findings were obtained in the challenge task with an accelerating rod from 4–40 rpm to 8–80 rpm, reflecting the robustness of the phenomenon. Evidence for muscular hypotonia was also obtained in other relevant assays by Chen et al. [38]. In humans, muscular hypotonia but also motor deficits, such as ataxic gait, clumsiness, and problems with balance, were repeatedly observed in individuals carrying *MYT1L* mutations [1–4, 11, 16, 18–20, 22].

Anxiety-related behavior was also altered. While the time spent in the center of the open field was not affected by *Myt1l* haploinsufficiency, the time spent in the open arms of the elevated plus maze was enhanced, reflecting reduced anxiety-related behavior. The effect is not driven by hyperactivity because the number of arm entries did not differ between genotypes. In humans, little is still known about the effects of *MYT1L* mutations on anxiety levels. While there are a few case studies reporting increased anxiety [14, 17], most studies do not report altered anxiety levels and others report happy demeanor with frequent smiling and outbursts of laughter [22]. Moreover, *Myt1l* haploinsufficiency led to enhanced acoustic startle reactivity, in line with findings obtained in the other mouse model for *Myt1l* haploinsufficiency [38]. Our results, however, suggest that this effect was sex-dependent. In males but not females, prominent genotype differences were observed and startle responses were potentiated in *Myt1l* heterozygous mice during the exposure to pulses with moderate and high sound intensities. Pre-pulse inhibition of acoustic startle was not affected by *Myt1l* haploinsufficiency irrespective of sex, suggesting intact sensorimotor gating, in line with the other *Myt1l* haploinsufficiency mouse model [38]. In light of the reduced anxiety-related behavior in the elevated plus maze, it appears unlikely that enhanced acoustic startle reactivity is driven by a stronger fear response to loud sound. Higher sensitivity to loud sound is thus favored as causal mechanism. In fact, albeit rarely assessed in humans, increased noise sensitivity was reported before in individuals carrying *MYT1L* mutations in a number of independent studies [2–4].

In contrast, learning and memory were only mildly affected by *Myt1l* haploinsufficiency and most pronounced effects were seen during fear conditioning, consistent with recent observations made in the other mouse model for *Myt1l* haploinsufficiency [38]. Firstly, spatial working memory in the Y-maze was intact. Likewise, there were no prominent effects on spatial and reversal learning abilities in the Barnes maze, albeit performance was slightly reduced in *Myt1l* heterozygous mice. Most notably, the affinity for the reversal target hole was higher compared to the initial target hole during reversal learning in wild-type littermates but did not reach statistical significance in *Myt1l* heterozygous mice. While this suggests mild effects of *Myt1l* haploinsufficiency, a different pattern with more prominent genotype differences was evident during fear conditioning. Genotype differences emerged with repeated tone-shock pairings and were most prominent toward the end of acquisition. In both sexes, *Myt1l* heterozygous mice displayed less freezing behavior than wild-type littermates. When placed back into the original conditioning chamber 24 h after acquisition, *Myt1l* heterozygous mice displayed less freezing behavior during contextual recall than wild-type littermates. This effect was particularly prominent in males, while not as clear in females, possibly because of lower freezing levels in females than in males. In contrast to contextual recall, cued recall was not affected by *Myt1l* haploinsufficiency. This suggests that *Myt1l* haploinsufficiency has a negative impact on contextual fear memory retrieval, while cued fear memory retrieval appears to be intact. Given the increased levels of locomotor activity together with the lower levels of anxiety-related behavior displayed by *Myt1l* heterozygous mice, however, it appears possible that this led to a reduction in freezing behavior. Freezing might be confounded by hyperactivity and/or low levels of anxiety. However, the fact that freezing behavior did not differ between *Myt1l* heterozygous mice and wild-type littermates during cued recall in an altered context speaks against a potential confound.

Finally, social behavior and socio-affective communication through ultrasonic vocalizations in adulthood appeared mostly unaffected by *Myt1l* haploinsufficiency. Although the sender emitting ultrasonic vocalizations could not be determined during social interactions of pairs, their emission seemed intact. During male–female social interactions, emission of ultrasonic vocalizations was high in pairs irrespective of the genotype of the male mouse. Relatively low levels of ultrasonic vocalizations were seen during female–female social interactions, and there was no evidence for genotype differences. In the three-chamber assay, social approach behavior was seen in *Myt1l* heterozygous mice and wild-type littermates. Nest building activities were enhanced in *Myt1l* heterozygous mice, resulting in higher nest quality ratings. Overall, this is mostly consistent with the findings obtained by Chen et al. [38]. There, mild deficits in social behavior were detected, such as reduced sociability in the three-chamber social approach assay and more submissive behavior in the social dominance tube test. Social novelty preference, however, was reported to be intact, and social reward seeking, i.e., nose-poking for getting access to a conspecific, was evident irrespective of genotype.

In summary, our findings in the newly generated mouse model for *Myt1l* haploinsufficiency are consistent with key elements of the clinical phenotype reported in humans, most notably obesity, hyperactivity, and reduced motor performance. However, the lack of prominent behavioral alterations with relevance to human ASD core symptoms might appear surprising. In humans, *MYT1L* loss-of-function mutations were repeatedly associated with ASD and *MYT1L* is ranked as a top ASD candidate gene [5–10]. A recent overview over 50 known individuals carrying *MYT1L* mutations reports that about 40% of them were diagnosed with ASD or displayed relevant phenotypes without formal diagnosis [4]. When focusing their analysis on mutations affecting *MYT1L* only, i.e., on microdeletions and single-nucleotide polymorphisms, the number reached almost 70%, while it was roughly 20% for larger microdeletions not only affecting *MYT1L*. In mice, in contrast, core domains of ASD were only mildly affected. Firstly, maternal odor preference in the homing test and social approach in the three-chamber test were intact. Nest building was enhanced. Secondly, socio-affective communication through ultrasonic vocalizations was mildly affected in pups but not in adulthood. Thirdly, no evidence for repetitive and stereotyped patterns of behavior was evident. Self-grooming behavior was not affected and reversal learning abilities appeared to be intact, despite minor evidence for worse performance. Finally, higher sensitivity to loud sound might be relevant in the context of ASD.

### Limitations

In future studies, a detailed characterization of direct reciprocal social interaction behavior might help to reveal effects of *Myt1l* haploinsufficiency on social behavior in juvenile and adult mice because this provides increased sensitivity and higher ethological validity than the three-chamber assay [48]. Given that *Myt1l* haploinsufficiency affected call clustering but not call emission rates, detailed spectrographic analyses of ultrasonic vocalizations emitted by adult mice might be beneficial [63]. Extending the analysis of repetitive behaviors by including other types of behaviors in addition might be beneficial as well [64]. Finally, more conservative statistical approaches need to be applied in future confirmatory studies, albeit the fact that the behavioral phenotypes obtained in the two independently generated mouse models for *Myt1l* haploinsufficiency are consistent speaks for the robustness of the findings [38].

## Summary and conclusions

Myt1l is not needed for neuronal specification but is essential for normal brain function and organismal survival. *Myt1l* haploinsufficiency led to obesity and multifaceted behavioral alterations in mice. In mouse pups, *Myt1l* haploinsufficiency caused mild alterations in early socio-affective communication, while social odor processing, social motivation, and basic motor functions appeared to be intact. In adults, *Myt1l* heterozygous mice displayed hyperactivity due to impaired habituation learning. Motor performance was reduced in *Myt1l* heterozygous mice despite intact motor learning, possibly due to muscular hypotonia. While anxiety-related behavior was reduced, acoustic startle reactivity was enhanced, in line with higher sensitivity to loud sound. Pre-pulse inhibition of acoustic startle was not altered, suggesting intact sensorimotor gating. Social behavior and socio-affective communication appeared to be intact during direct reciprocal social interactions in adult mice. Repetitive behavior quantified through self-grooming was not evident. Nest building activities were enhanced. Spatial memory and reversal learning appeared to be intact. *Myt1l* haploinsufficiency had a negative impact on contextual fear memory retrieval, while cued fear memory retrieval appeared to be intact. Despite smaller inconsistencies, the consistency across the two recently generated mouse models for *Myt1l* haploinsufficiency is remarkable. Most of the behavioral phenotypes replicate—and importantly, behavioral alterations in heterozygous, loss-of-function Myt1l mice recapitulate several clinical phenotypes observed in humans carrying *MYT1*L mutations and thus serve as an informative model of the human *MYT1L* syndrome.

## Supplementary Information

**Additional file 1. Figure S1: **Molecular characterization of Myt1l expression in mutant mice. (A) Western blot analysis of Myt1l expression in WT or *Myt1l* HET mouse embryo brains. RabGDI was used as a loading control. (B) Quantification of Myt1l protein expression. The Myt1l bands signal intensity was normalized to that of RabGDI and then to the value of 1.0 for WT (N=3/genotype, one sample t test; P=0.022; error bar, s.d.). (C) qRT-PCR measuring Myt1l transcript levels in brains of WT and HET males (16 month old), normalized to MAP2 (N=3/genotype, t test; ns, not significant, P>0.05; error bars, s.d.). (D) Uncropped Western blot of the blot shown in Suppl. Figure S1A. (E) Uncropped Western blot of the blot shown in Figure 1C.

**Additional file 2. Figure S2: **No overt morphological differences between wild-type and *Myt1l* mutant mouse brains. Nissl-stained sections from three animal triplets (*Myt1l*^+/+^, *Myt1l*^+/-^, *Myt1*^-/-^), aged E18.5, from three different litters (N=3/genotype). Examples depict slices corresponding to section 125 (rostral) and 155 (caudal) of the Allen Atlas of the Developing Mouse Brain.

**Additional file 3. Figure S3: **Effects of *Myt1l* haploinsufficiency on the emission of isolation-induced ultrasonic vocalizations in the homing test – Acoustic features. (A) Call duration is not altered in *Myt1l*^+/-^ mouse pups. ANOVA with the between-subject factors genotype (G) and sex (S); all p values > 0.050. (B) Peak frequency is unchanged in *Myt1l*^+/-^ mouse pups. ANOVA with the between-subject factors genotype (G) and sex (S); all p values > 0.050. (C) Frequency modulation is not affected in *Myt1l*^+/-^ mouse pups. ANOVA with the between-subject factors genotype (G) and sex (S); all p values > 0.050. (D) Peak amplitude is unchanged in *Myt1l*^+/-^ mouse pups. ANOVA with the between-subject factors genotype (G) and sex (S); all p values > 0.050. All data are means ± SEM, combined across males and females.

**Additional file 4. Figure S4: **Effects of *Myt1l* haploinsufficiency on the emission of isolation-induced ultrasonic vocalizations in the homing test – Temporal Organization. (A-C) Sequential analysis of the durations of subsequent isolation-induced ultrasonic vocalizations indicating a non-random call emission pattern in *Myt1l*^+/-^ mouse pups. Correlations between the durations of given isolation-induced USV and the durations of the previous ones (N – 1), the durations of the ones two before (N – 2), and the durations of the ones three before (N – 3). Sequential analysis is based on individual isolation-induced ultrasonic vocalizations in *Myt1l*^+/+^ littermate controls (~20,000 calls) and *Myt1l*^+/-^ mouse pups (~30,000 calls), combined across males and females. # = p < 0.05 vs. correlation coefficient r = 0.

**Additional file 5. Figure S5: **Effects of *Myt1l* haploinsufficiency on fear conditioning. (A-C) Fear-related freezing behavior is unchanged in male and female *Myt1l*^+/-^ mice during cued recall. Gray highlighting indicates tone presentations. Repeated-measures ANOVAs with the between-subject factors genotype (G) and sex (S) and the within-subject factor time (T). T: F_11,572_=12.667; p<.001; TxS: F_11,572_=1.996; p=.027; all other p values > 0.050. All data are means ± SEM, combined across males and females if not otherwise indicated.

**Additional file 6. Table S1:** Overview on statistical results for behavioral assays.

## Data Availability

All data are available in the manuscript or supplementary material. For requests on reagents and mice, please contact M.We. at wernig@stanford.edu. For information on behavioral assays, please contact M.Wö. at markus.wohr@kuleuven.be.

## References

[1] Blanchet P, Bebin M, Bruet S, Cooper GM, Thompson ML, Duban-Bedu B, Gerard B, Piton A, Suckno S, Deshpande C, Clowes V, Vogt J, Turnpenny P, Williamson MP, Alembik Y. MYT1L mutations cause intellectual disability and variable obesity by dysregulating gene expression and development of the neuroendocrine hypothalamus. PLoS Genet. 2017;13(8):e1006957. 10.1371/journal.pgen.1006957. 28859103 10.1371/journal.pgen.1006957 PMC5597252

[2] De Rocker N, Vergult S, Koolen D, Jacobs E, Hoischen A, Zeesman S, Bang B, Béna F, Bockaert N, Bongers EM, deRavel T, Devriendt K, Giglio S, Faivre L, Joss S, Maas S, Marle N, Novara F, Nowaczyk MJ, Peeters H, Polstra A, Roelens F, Rosenberg C, Thevenon J, Tümer Z, Vanhauwaert S, Varvagiannis K, Willaert A, Willemsen M, Willems M, Zuffardi O, Coucke P, Speleman F, Eichler EE, Kleefstra T, Menten B. Refinement of the critical 2p25.3 deletion region: the role of MYT1L in intellectual disability and obesity. Genet Med. 2015;17(6):460–6. 10.1038/gim.2014.124. 25232846 10.1038/gim.2014.124

[3] Stevens SJ, van Ravenswaaij-Arts CM, Janssen JW, Klein Wassink-Ruiter JS, van Essen AJ, Dijkhuizen T, van Rheenen J, Heuts-Vijgen R, Stegmann AP, Smeets EE, Engelen JJ. MYT1L is a candidate gene for intellectual disability in patients with 2p25.3 (2pter) deletions. Am J Med Genet A. 2011;155A(11):2739–45. 10.1002/ajmg.a.34274. 21990140 10.1002/ajmg.a.34274

[4] Windheuser IC, Becker J, Cremer K, Hundertmark H, Yates LM, Mangold E, Peters S, Degenhardt F, Ludwig KU, Zink AM, Lessel D, Bierhals T, Herget T, Johannsen J, Denecke J, Wohlleber E, Strom TM, Wieczorek D, Bertoli M, Colombo R, Hempel M, Engels H. Nine newly identified individuals refine the phenotype associated with MYT1L mutations. Am J Med Genet A. 2020;182(5):1021–31. 10.1002/ajmg.a.61515. 32065501 10.1002/ajmg.a.61515

[5] Satterstrom FK, Kosmicki JA, Wang J, Breen MS, De Rubeis S, An JY, Peng M, Collins R, Grove J, Klei L, Stevens C, Reichert J, Mulhern MS, Artomov M, Gerges S, Sheppard B, Xu X, Bhaduri A, Norman U, Brand H, Schwartz G, Nguyen R, Guerrero EE, Dias C; Autism Sequencing Consortium; iPSYCH-Broad Consortium, Betancur C, Cook EH, Gallagher L, Gill M, Sutcliffe JS, Thurm A, Zwick ME, Børglum AD, State MW, Cicek AE, Talkowski ME, Cutler DJ, Devlin B, Sanders SJ, Roeder K, Daly MJ, Buxbaum JD. Large-Scale Exome Sequencing Study Implicates Both Developmental and Functional Changes in the Neurobiology of Autism. Cell. 2020;180(3):568–584.e23. 10.1016/j.cell.2019.12.036. 10.1016/j.cell.2019.12.036 PMC7250485 31981491

[6] Deciphering Developmental Disorders Study. Prevalence and architecture of de novo mutations in developmental disorders. Nature. 2017;542(7642):433–438. 10.1038/nature21062. 10.1038/nature21062 PMC6016744 28135719

[7] De Rubeis S, He X, Goldberg AP, Poultney CS, Samocha K, Cicek AE, Kou Y, Liu L, Fromer M, Walker S, Singh T, Klei L, Kosmicki J, Shih-Chen F, Aleksic B, Biscaldi M, Bolton PF, Brownfeld JM, Cai J, Campbell NG, Carracedo A, Chahrour MH, Chiocchetti AG, Coon H, Crawford EL, Curran SR, Dawson G, Duketis E, Fernandez BA, Gallagher L, Geller E, Guter SJ, Hill RS, Ionita-Laza J, Jimenz Gonzalez P, Kilpinen H, Klauck SM, Kolevzon A, Lee I, Lei I, Lei J, Lehtimäki T, Lin CF, Ma'ayan A, Marshall CR, McInnes AL, Neale B, Owen MJ, Ozaki N, Parellada M, Parr JR, Purcell S, Puura K, Rajagopalan D, Rehnström K, Reichenberg A, Sabo A, Sachse M, Sanders SJ, Schafer C, Schulte-Rüther M, Skuse D, Stevens C, Szatmari P, Tammimies K, Valladares O, Voran A, Li-San W, Weiss LA, Willsey AJ, Yu TW, Yuen RK; DDD Study; Homozygosity Mapping Collaborative for Autism; UK10K Consortium, Cook EH, Freitag CM, Gill M, Hultman CM, Lehner T, Palotie A, Schellenberg GD, Sklar P, State MW, Sutcliffe JS, Walsh CA, Scherer SW, Zwick ME, Barett JC, Cutler DJ, Roeder K, Devlin B, Daly MJ, Buxbaum JD. Synaptic, transcriptional and chromatin genes disrupted in autism. Nature. 2014;515(7526):209–15. 10.1038/nature13772. 10.1038/nature13772 PMC4402723 25363760

[8] Sanders SJ, He X, Willsey AJ, Ercan-Sencicek AG, Samocha KE, Cicek AE, Murtha MT, Bal VH, Bishop SL, Dong S, Goldberg AP, Jinlu C, Keaney JF 3rd, Klei L, Mandell JD, Moreno-De-Luca D, Poultney CS, Robinson EB, Smith L, Solli-Nowlan T, Su MY, Teran NA, Walker MF, Werling DM, Beaudet AL, Cantor RM, Fombonne E, Geschwind DH, Grice DE, Lord C, Lowe JK, Mane SM, Martin DM, Morrow EM, Talkowski ME, Sutcliffe JS, Walsh CA, Yu TW; Autism Sequencing Consortium, Ledbetter DH, Martin CL, Cook EH, Buxbaum JD, Daly MJ, Devlin B, Roeder K, State MW. Insights into Autism Spectrum Disorder Genomic Architecture and Biology from 71 Risk Loci. Neuron. 2015;87(6):1215–1233. 10.1016/j.neuron.2015.09.016. 10.1016/j.neuron.2015.09.016 PMC4624267 26402605

[9] Wang T, Guo H, Xiong B, Stessman HA, Wu H, Coe BP, Turner TN, Liu Y, Zhao W, Hoekzema K, Vives L, Xia L, Tang M, Ou J, Chen B, Shen Y, Xun G, Long M, Lin J, Kronenberg ZN, Peng Y, Bai T, Li H, Ke X, Hu Z, Zhao J, Zou X, Xia K, Eichler EE. De novo genic mutations among a Chinese autism spectrum disorder cohort. Nat Commun. 2016;8(7):13316. 10.1038/ncomms13316. 10.1038/ncomms13316 PMC5105161 27824329

[10] Yuen RKC, Merico D, Bookman M, L Howe J, Thiruvahindrapuram B, Patel RV, Whitney J, Deflaux N, Bingham J, Wang Z, Pellecchia G, Buchanan JA, Walker S, Marshall CR, Uddin M, Zarrei M, Deneault E, D'Abate L, Chan AJ, Koyanagi S, Paton T, Pereira SL, Hoang N, Engchuan W, Higginbotham EJ, Ho K, Lamoureux S, Li W, MacDonald JR, Nalpathamkalam T, Sung WW, Tsoi FJ, Wei J, Xu L, Tasse AM, Kirby E, Van Etten W, Twigger S, Roberts W, Drmic I, Jilderda S, Modi BM, Kellam B, Szego M, Cytrynbaum C, Weksberg R, Zwaigenbaum L, Woodbury-Smith M, Brian J, Senman L, Iaboni A, Doyle-Thomas K, Thompson A, Chrysler C, Leef J, Savion-Lemieux T, Smith IM, Liu X, Nicolson R, Seifer V, Fedele A, Cook EH, Dager S, Estes A, Gallagher L, Malow BA, Parr JR, Spence SJ, Vorstman J, Frey BJ, Robinson JT, Strug LJ, Fernandez BA, Elsabbagh M, Carter MT, Hallmayer J, Knoppers BM, Anagnostou E, Szatmari P, Ring RH, Glazer D, Pletcher MT, Scherer SW. Whole genome sequencing resource identifies 18 new candidate genes for autism spectrum disorder. Nat Neurosci. 2017; 20(4):602–611. 10.1038/nn.4524. 10.1038/nn.4524 PMC5501701 28263302

[11] Al Tuwaijri A, Alfadhel M. MYT1L mutation in a patient causes intellectual disability and early onset of obesity: a case report and review of the literature. J Pediatr Endocrinol Metab. 2019;32(4):409–13. 10.1515/jpem-2018-0505. 30796847 10.1515/jpem-2018-0505

[12] Bonaglia MC, Giorda R, Zanini S. A new patient with a terminal de novo 2p25.3 deletion of 1.9 Mb associated with early-onset of obesity, intellectual disabilities and hyperkinetic disorder. Mol Cytogenet. 2014;7:53. 10.1186/1755-8166-7-53. 25126114 10.1186/1755-8166-7-53 PMC4131807

[13] Braddock A, Del Campo M, Reiff MI, Stein MT. Disruptive behavior, global developmental delay, and obesity in a 5-year-old boy with a chromosome microduplication. J Dev Behav Pediatr. 2018;39(1):81–4. 10.1097/DBP.0000000000000528. 29293472 10.1097/DBP.0000000000000528

[14] Carvalho LML, D’Angelo CS, Mustacchi Z, da Silva IT, Krepischi ACV, Koiffmann CP, Rosenberg C. A novel MYT1L mutation in a boy with syndromic obesity: case report and literature review. Obes Res Clin Pract. 2021;15(2):124–32. 10.1016/j.orcp.2021.01.001. 33622623 10.1016/j.orcp.2021.01.001

[15] D’Angelo CS, Varela MC, de Castro CIE, Otto PA, Perez ABA, Lourenço CM, Kim CA, Bertola DR, Kok F, Garcia-Alonso L, Koiffmann CP. Chromosomal microarray analysis in the genetic evaluation of 279 patients with syndromic obesity. Mol Cytogenet. 2018;5(11):14. 10.1186/s13039-018-0363-7. 10.1186/s13039-018-0363-7 PMC5800070 29441128

[16] de Ligt J, Willemsen MH, van Bon BW, Kleefstra T, Yntema HG, Kroes T, Vulto-van Silfhout AT, Koolen DA, de Vries P, Gilissen C, del Rosario M, Hoischen A, Scheffer H, de Vries BB, Brunner HG, Veltman JA, Vissers LE. Diagnostic exome sequencing in persons with severe intellectual disability. N Engl J Med. 2012;367(20):1921–9. 10.1056/NEJMoa1206524. 23033978 10.1056/NEJMoa1206524

[17] Doco-Fenzy M, Leroy C, Schneider A, Petit F, Delrue MA, Andrieux J, Perrin-Sabourin L, Landais E, Aboura A, Puechberty J, Girard M, Tournaire M, Sanchez E, Rooryck C, Ameil A, Goossens M, Jonveaux P, Lefort G, Taine L, Cailley D, Gaillard D, Leheup B, Sarda P, Geneviève D. Early-onset obesity and paternal 2pter deletion encompassing the ACP1, TMEM18, and MYT1L genes. Eur J Hum Genet. 2014;22(4):471–9. 10.1038/ejhg.2013.189. 24129437 10.1038/ejhg.2013.189 PMC3953915

[18] Loid P, Mäkitie R, Costantini A, Viljakainen H, Pekkinen M, Mäkitie O. A novel MYT1L mutation in a patient with severe early-onset obesity and intellectual disability. Am J Med Genet A. 2018;176(9):1972–5. 10.1002/ajmg.a.40370. 30055078 10.1002/ajmg.a.40370

[19] Loid P, Mustila T, Mäkitie RE, Viljakainen H, Kämpe A, Tossavainen P, Lipsanen-Nyman M, Pekkinen M, Mäkitie O. Rare variants in genes linked to appetite control and hypothalamic development in early-onset severe obesity. Front Endocrinol (Lausanne). 2020;21(11):81. 10.3389/fendo.2020.00081. 10.3389/fendo.2020.00081 PMC7047210 32153512

[20] Mayo S, Roselló M, Monfort S, Oltra S, Orellana C, Martínez F. Haploinsufficiency of the MYT1L gene causes intellectual disability frequently associated with behavioral disorder. Genet Med. 2015;17(8):683–4. 10.1038/gim.2015.86. 26240977 10.1038/gim.2015.86

[21] Meyer KJ, Axelsen MS, Sheffield VC, Patil SR, Wassink TH. Germline mosaic transmission of a novel duplication of PXDN and MYT1L to two male half-siblings with autism. Psychiatr Genet. 2012;22(3):137–40. 10.1097/YPG.0b013e32834dc3f5. 22157634 10.1097/YPG.0b013e32834dc3f5 PMC3309069

[22] Rio M, Royer G, Gobin S, de Blois MC, Ozilou C, Bernheim A, Nizon M, Munnich A, Bonnefont JP, Romana S, Vekemans M, Turleau C, Malan V. Monozygotic twins discordant for submicroscopic chromosomal anomalies in 2p25.3 region detected by array CGH. Clin Genet. 2013;84(1):31–6. 10.1111/cge.12036. 23061379 10.1111/cge.12036

[23] Lee Y, Mattai A, Long R, Rapoport JL, Gogtay N, Addington AM. Microduplications disrupting the MYT1L gene (2p25.3) are associated with schizophrenia. Psychiatr Genet. 2012;22(4):206–9. 10.1097/YPG.0b013e328353ae3d. 22547139 10.1097/YPG.0b013e328353ae3d PMC3384746

[24] Van Den Bossche MJ, Strazisar M, Cammaerts S, Liekens AM, Vandeweyer G, Depreeuw V, Mattheijssens M, Lenaerts AS, De Zutter S, De Rijk P, Sabbe B, Del-Favero J. Identification of rare copy number variants in high burden schizophrenia families. Am J Med Genet B Neuropsychiatr Genet. 2013;162B(3):273–82. 10.1002/ajmg.b.32146. 23505263 10.1002/ajmg.b.32146

[25] Vrijenhoek T, Buizer-Voskamp JE, van der Stelt I, Strengman E; Genetic Risk and Outcome in Psychosis (GROUP) Consortium, Sabatti C, Geurts van Kessel A, Brunner HG, Ophoff RA, Veltman JA. Recurrent CNVs disrupt three candidate genes in schizophrenia patients. Am J Hum Genet. 2008;83(4):504–10. 10.1016/j.ajhg.2008.09.011. 10.1016/j.ajhg.2008.09.011 PMC2561936 18940311

[26] Li W, Wang X, Zhao J, Lin J, Song XQ, Yang Y, Jiang C, Xiao B, Yang G, Zhang HX, Lv LX. Association study of myelin transcription factor 1-like polymorphisms with schizophrenia in Han Chinese population. Genes Brain Behav. 2012;11(1):87–93. 10.1111/j.1601-183X.2011.00734.x. 21923761 10.1111/j.1601-183X.2011.00734.x

[27] Mansfield P, Constantino JN, Baldridge D. MYT1L: a systematic review of genetic variation encompassing schizophrenia and autism. Am J Med Genet B Neuropsychiatr Genet. 2020;183(4):227–33. 10.1002/ajmg.b.32781. 32267091 10.1002/ajmg.b.32781 PMC7605444

[28] Jiang Y, Yu VC, Buchholz F, O’Connell S, Rhodes SJ, Candeloro C, Xia YR, Lusis AJ, Rosenfeld MG. A novel family of Cys-Cys, His-Cys zinc finger transcription factors expressed in developing nervous system and pituitary gland. J Biol Chem. 1996;271(18):10723–30. 10.1074/jbc.271.18.10723. 8631881 10.1074/jbc.271.18.10723

[29] Kim JG, Armstrong RC, Agoston D, Robinsky A, Wiese C, Nagle J, Hudson LD. Myelin transcription factor 1 (Myt1) of the oligodendrocyte lineage, along with a closely related CCHC zinc finger, is expressed in developing neurons in the mammalian central nervous system. J Neurosci Res. 1997;50(2):272–90. 9373037 10.1002/(SICI)1097-4547(19971015)50:2<272::AID-JNR16>3.0.CO;2-A

[30] Weiner JA, Chun J. Png-1, a nervous system-specific zinc finger gene, identifies regions containing postmitotic neurons during mammalian embryonic development. J Comp Neurol. 1997;381(2):130–42. 10.1002/(sici)1096-9861(19970505)381:2%3c130::aid-cne2%3e3.0.co;2-4. 9130664 10.1002/(sici)1096-9861(19970505)381:2<130::aid-cne2>3.0.co;2-4

[31] Matsushita F, Kameyama T, Kadokawa Y, Marunouchi T. Spatiotemporal expression pattern of Myt/NZF family zinc finger transcription factors during mouse nervous system development. Dev Dyn. 2014;243(4):588–600. 10.1002/dvdy.24091. 24214099 10.1002/dvdy.24091

[32] Pang ZP, Yang N, Vierbuchen T, Ostermeier A, Fuentes DR, Yang TQ, Citri A, Sebastiano V, Marro S, Südhof TC, Wernig M. Induction of human neuronal cells by defined transcription factors. Nature. 2011;476(7359):220–3. 10.1038/nature10202. 21617644 10.1038/nature10202 PMC3159048

[33] Vierbuchen T, Ostermeier A, Pang ZP, Kokubu Y, Südhof TC, Wernig M. Direct conversion of fibroblasts to functional neurons by defined factors. Nature. 2010;463(7284):1035–41. 10.1038/nature08797. 20107439 10.1038/nature08797 PMC2829121

[34] Chanda S, Ang CE, Davila J, Pak C, Mall M, Lee QY, Ahlenius H, Jung SW, Südhof TC, Wernig M. Generation of induced neuronal cells by the single reprogramming factor ASCL1. Stem Cell Rep. 2014;3(2):282–96. 10.1016/j.stemcr.2014.05.020. 10.1016/j.stemcr.2014.05.020 PMC4176533 25254342

[35] Treutlein B, Lee QY, Camp JG, Mall M, Koh W, Shariati SA, Sim S, Neff NF, Skotheim JM, Wernig M, Quake SR. Dissecting direct reprogramming from fibroblast to neuron using single-cell RNA-seq. Nature. 2016;534(7607):391–5. 10.1038/nature18323. 27281220 10.1038/nature18323 PMC4928860

[36] Mall M, Kareta MS, Chanda S, Ahlenius H, Perotti N, Zhou B, Grieder SD, Ge X, Drake S, Euong Ang C, Walker BM, Vierbuchen T, Fuentes DR, Brennecke P, Nitta KR, Jolma A, Steinmetz LM, Taipale J, Südhof TC, Wernig M. Myt1l safeguards neuronal identity by actively repressing many non-neuronal fates. Nature. 2017;544(7649):245–9. 10.1038/nature21722. 28379941 10.1038/nature21722 PMC11348803

[37] Chong JA, Tapia-Ramírez J, Kim S, Toledo-Aral JJ, Zheng Y, Boutros MC, Altshuller YM, Frohman MA, Kraner SD, Mandel G. REST: a mammalian silencer protein that restricts sodium channel gene expression to neurons. Cell. 1995;80(6):949–57. 10.1016/0092-8674(95)90298-8. 7697725 10.1016/0092-8674(95)90298-8

[38] Chen J, Lambo ME, Ge X, Dearborn JT, Liu Y, McCullough KB, Swift RG, Tabachnick DR, Tian L, Noguchi K, Garbow JR, Constantino JN, Gabel HW, Hengen KB, Maloney SE, Dougherty JD. A MYT1L syndrome mouse model recapitulates patient phenotypes and reveals altered brain development due to disrupted neuronal maturation. Neuron. 2021;109(23):3775-3792.e14. 10.1016/j.neuron.2021.09.009. 34614421 10.1016/j.neuron.2021.09.009 PMC8668036

[39] Eenjes E, Dragich JM, Kampinga HH, Yamamoto A. Distinguishing aggregate formation and aggregate clearance using cell-based assays. J Cell Sci. 2016;129(6):1260–70. 10.1242/jcs.179978. 26818841 10.1242/jcs.179978 PMC4813294

[40] Oo TF, Burke RE. Apoptotic morphology in phenotypically defined dopaminergic neurons of the substantia nigra. Methods Mol Med. 2001;62:101–12. 10.1385/1-59259-142-6:101. 21318771 10.1385/1-59259-142-6:101

[41] Silverman JL, Thurm A, Ethridge SB, Soller MM, Petkova SP, Abel T, Bauman MD, Brodkin ES, Harony-Nicolas H, Wöhr M, Halladay A. Reconsidering animal models used to study autism spectrum disorder: current state and optimizing future. Genes Brain Behav. 2022;14:e12803. 10.1111/gbb.12803. 10.1111/gbb.12803 PMC9189007 35285132

[42] Silverman JL, Yang M, Lord C, Crawley JN. Behavioural phenotyping assays for mouse models of autism. Nat Rev Neurosci. 2010;11(7):490–502. 10.1038/nrn2851. 20559336 10.1038/nrn2851 PMC3087436

[43] Wöhr M, Scattoni ML. Behavioural methods used in rodent models of autism spectrum disorders: current standards and new developments. Behav Brain Res. 2013;15(251):5–17. 10.1016/j.bbr.2013.05.047. 10.1016/j.bbr.2013.05.047 23769995

[44] Scattoni ML, Gandhy SU, Ricceri L, Crawley JN. Unusual repertoire of vocalizations in the BTBR T+tf/J mouse model of autism. PLoS ONE. 2008;3(8):e3067. 10.1371/journal.pone.0003067. 18728777 10.1371/journal.pone.0003067 PMC2516927

[45] Rothwell PE, Fuccillo MV, Maxeiner S, Hayton SJ, Gokce O, Lim BK, Fowler SC, Malenka RC, Südhof TC. Autism-associated neuroligin-3 mutations commonly impair striatal circuits to boost repetitive behaviors. Cell. 2014;158(1):198–212. 10.1016/j.cell.2014.04.045. 24995986 10.1016/j.cell.2014.04.045 PMC4120877

[46] Zhou M, Liu Z, Melin MD, Ng YH, Xu W, Südhof TC. A central amygdala to zona incerta projection is required for acquisition and remote recall of conditioned fear memory. Nat Neurosci. 2018;21(11):1515–9. 10.1038/s41593-018-0248-4. 30349111 10.1038/s41593-018-0248-4 PMC7261007

[47] Sungur AÖ, Jochner MCE, Harb H, Kılıç A, Garn H, Schwarting RKW, Wöhr M. Aberrant cognitive phenotypes and altered hippocampal BDNF expression related to epigenetic modifications in mice lacking the post-synaptic scaffolding protein SHANK1: implications for autism spectrum disorder. Hippocampus. 2017;27(8):906–19. 10.1002/hipo.22741. 28500650 10.1002/hipo.22741

[48] Wöhr M, Orduz D, Gregory P, Moreno H, Khan U, Vörckel KJ, Wolfer DP, Welzl H, Gall D, Schiffmann SN, Schwaller B. Lack of parvalbumin in mice leads to behavioral deficits relevant to all human autism core symptoms and related neural morphofunctional abnormalities. Transl Psychiatry. 2015;5(3):e525. 10.1038/tp.2015.19. 25756808 10.1038/tp.2015.19 PMC4354349

[49] Etherton MR, Blaiss CA, Powell CM, Südhof TC. Mouse neurexin-1alpha deletion causes correlated electrophysiological and behavioral changes consistent with cognitive impairments. Proc Natl Acad Sci USA. 2009;106(42):17998–8003. 10.1073/pnas.0910297106. 19822762 10.1073/pnas.0910297106 PMC2764944

[50] Dai J, Aoto J, Südhof TC. Alternative splicing of presynaptic neurexins differentially controls postsynaptic NMDA and AMPA receptor responses. Neuron. 2019;102(5):993-1008.e5. 10.1016/j.neuron.2019.03.032. 31005376 10.1016/j.neuron.2019.03.032 PMC6554035

[51] Modzelewski AJ, Chen S, Willis BJ, Lloyd KCK, Wood JA, He L. Efficient mouse genome engineering by CRISPR-EZ technology. Nat Protoc. 2018;13(6):1253–74. 10.1038/nprot.2018.012. 29748649 10.1038/nprot.2018.012 PMC6296855

[52] Leibowitz SF, Hammer NJ, Chang K. Hypothalamic paraventricular nucleus lesions produce overeating and obesity in the rat. Physiol Behav. 1981;27(6):1031–40. 10.1016/0031-9384(81)90366-8. 7335803 10.1016/0031-9384(81)90366-8

[53] Wöhr M, Schwarting RK. Affective communication in rodents: ultrasonic vocalizations as a tool for research on emotion and motivation. Cell Tissue Res. 2013;354(1):81–97. 10.1007/s00441-013-1607-9. 23576070 10.1007/s00441-013-1607-9

[54] Liu RC, Schreiner CE. Auditory cortical detection and discrimination correlates with communicative significance. PLoS Biol. 2007;5(7):e173. 10.1371/journal.pbio.0050173. 17564499 10.1371/journal.pbio.0050173 PMC1891324

[55] Ehret G. Infant rodent ultrasounds: a gate to the understanding of sound communication. Behav Genet. 2005;35(1):19–29. 10.1007/s10519-004-0853-8. 15674530 10.1007/s10519-004-0853-8

[56] Caruso A, Ricceri L, Scattoni ML. Ultrasonic vocalizations as a fundamental tool for early and adult behavioral phenotyping of Autism Spectrum Disorder rodent models. Neurosci Biobehav Rev. 2020;116:31–43. 10.1016/j.neubiorev.2020.06.011. 32544538 10.1016/j.neubiorev.2020.06.011

[57] Wöhr M, Dahlhoff M, Wolf E, Holsboer F, Schwarting RK, Wotjak CT. Effects of genetic background, gender, and early environmental factors on isolation-induced ultrasonic calling in mouse pups: an embryo-transfer study. Behav Genet. 2008;38(6):579–95. 10.1007/s10519-008-9221-4. 18712592 10.1007/s10519-008-9221-4

[58] Fyke W, Premoli M, Echeverry Alzate V, López-Moreno JA, Lemaire-Mayo V, Crusio WE, Marsicano G, Wöhr M, Pietropaolo S. Communication and social interaction in the cannabinoid-type 1 receptor null mouse: implications for autism spectrum disorder. Autism Res. 2021. 10.1002/aur.2562. 34173729 10.1002/aur.2562

[59] Mosienko V, Beis D, Alenina N, Wöhr M. Reduced isolation-induced pup ultrasonic communication in mouse pups lacking brain serotonin. Mol Autism. 2015;8(6):13. 10.1186/s13229-015-0003-6. 10.1186/s13229-015-0003-6 PMC4404606 25901271

[60] Sungur AÖ, Schwarting RK, Wöhr M. Early communication deficits in the Shank1 knockout mouse model for autism spectrum disorder: developmental aspects and effects of social context. Autism Res. 2016;9(6):696–709. 10.1002/aur.1564. 26419918 10.1002/aur.1564

[61] Rankin CH, Abrams T, Barry RJ, Bhatnagar S, Clayton DF, Colombo J, Coppola G, Geyer MA, Glanzman DL, Marsland S, McSweeney FK, Wilson DA, Wu CF, Thompson RF. Habituation revisited: an updated and revised description of the behavioral characteristics of habituation. Neurobiol Learn Mem. 2009;92(2):135–8. 10.1016/j.nlm.2008.09.012. 18854219 10.1016/j.nlm.2008.09.012 PMC2754195

[62] McDiarmid TA, Bernardos AC, Rankin CH. Habituation is altered in neuropsychiatric disorders—a comprehensive review with recommendations for experimental design and analysis. Neurosci Biobehav Rev. 2017;80:286–305. 10.1016/j.neubiorev.2017.05.028. 28579490 10.1016/j.neubiorev.2017.05.028

[63] Holy TE, Guo Z. Ultrasonic songs of male mice. PLoS Biol. 2005;3(12):e386. 10.1371/journal.pbio.0030386. 16248680 10.1371/journal.pbio.0030386 PMC1275525

[64] Kalueff AV, Stewart AM, Song C, Berridge KC, Graybiel AM, Fentress JC. Neurobiology of rodent self-grooming and its value for translational neuroscience. Nat Rev Neurosci. 2016;17(1):45–59. 10.1038/nrn.2015.8. 26675822 10.1038/nrn.2015.8 PMC4840777

