# Correction to: *Myt1l* haploinsufficiency leads to obesity and multifaceted behavioral alterations in mice

**DOI:** 10.1186/s13229-025-00677-x

**Published:** 2025-08-22

**Authors:** Markus Wöhr, Wendy M. Fong, Justyna A. Janas, Moritz Mall, Christian Thome, Madhuri Vangipuram, Lingjun Meng, Thomas C. Südhof, Marius Wernig

**Affiliations:** 1 Department of Molecular and Cellular Physiology, School of Medicine, Stanford University, Stanford, CA 94305 USA; 2 Research Unit Brain and Cognition, Laboratory of Biological Psychology, Social and Affective Neuroscience Research Group, Faculty of Psychology and Educational Sciences, KU Leuven, Leuven, 3000 Belgium; 3 Leuven Brain Institute, KU Leuven, Leuven, 3000 Belgium; 4 Faculty of Psychology, Experimental and Biological Psychology, Behavioral Neuroscience, Philipps-University of Marburg, 35032 Marburg, Germany; 5 Center for Mind, Brain and Behavior, Philipps-University of Marburg, 35032 Marburg, Germany; 6 Departments of Pathology and Chemical and Systems Biology, School of Medicine, Institute for Stem Cell Biology and Regenerative Medicine, Stanford University, Stanford, CA 94305 USA; 7 School of Medicine, Howard Hughes Medical Institute, Stanford University, Stanford, CA 94305 USA; 8 Present Address: Cell Fate Engineering and Disease Modeling Group, German Cancer Research Center (DKFZ) and DKFZ-ZMBH Alliance, 69120 Heidelberg, Germany; 9 Present Address: HITBR Hector Institute for Translational Brain Research gGmbH, 69120 Heidelberg, Germany; 10 Present Address: Central Institute of Mental Health, Medical Faculty Mannheim, Heidelberg University, 68159 Mannheim, Germany

**Correction to: Molecular Autism 13, 19 (2022).** 10.1186/s13229-022-00497-3

In this article, the authors reported an error in Additional File 2, specifically in the heterozygous column, where duplicated images were inadvertently included for litters B and C in the rostral and caudal panels. These duplications were the result of copy-paste errors during the final figure assembly in PowerPoint and do not affect the original data or the validity of the study’s conclusions.

The incorrect and corrected image in Additional file 2 are included below and Additional file 2 in the original article has been updated.

**Incorrect image in Additional file 2**:

**Additional file 2. Figure S2:**
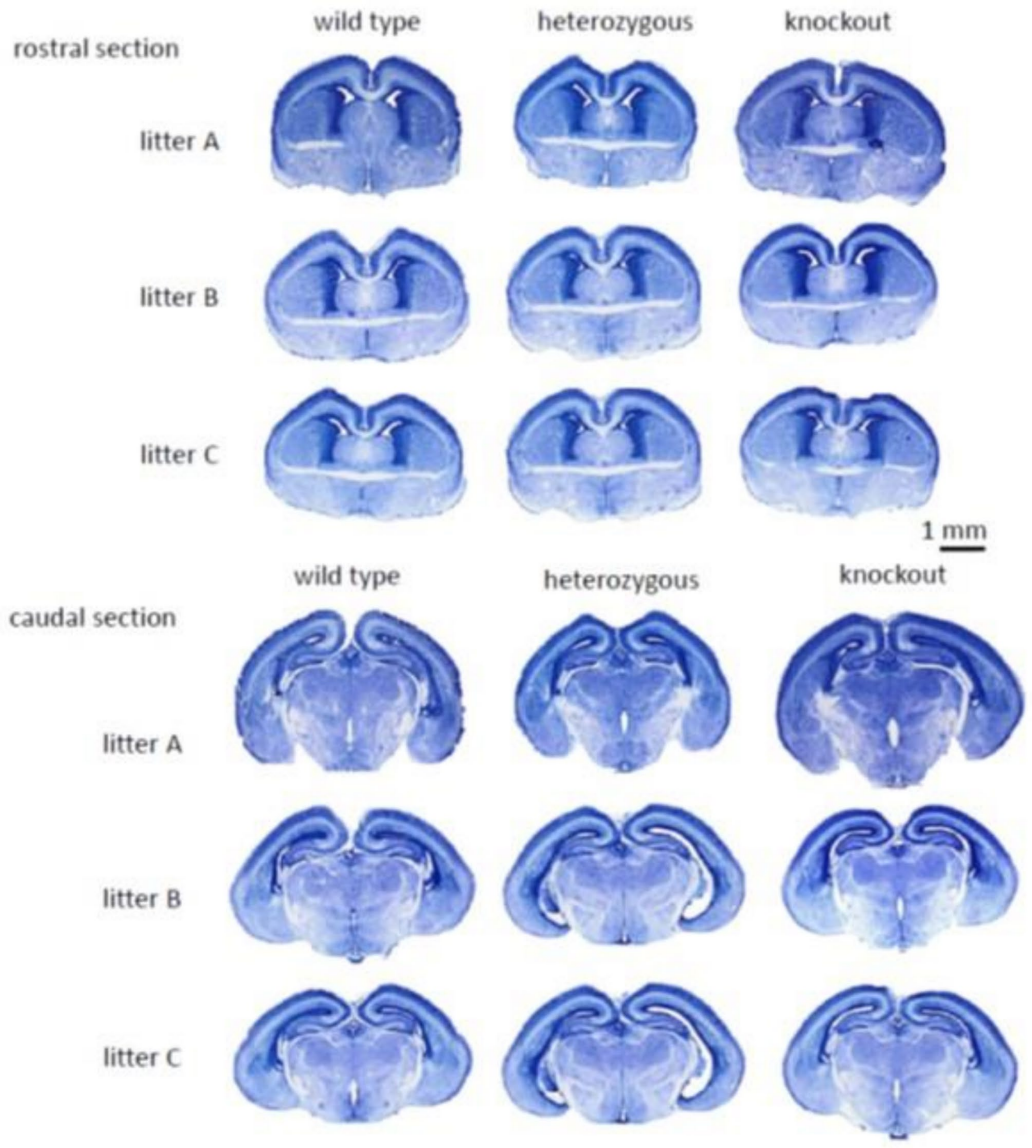
No overt morphological differences between wild-type and *Myt1l* mutant mouse brains. Nissl-stained sections from three animal triplets (*Myt1l*^+/+^, *Myt1l*^+/−^, *Myt1*^−/−^), aged E18.5, from three different litters (*N* = 3/genotype). Examples depict slices corresponding to section 125 (rostral) and 155 (caudal) of the Allen Atlas of the Developing Mouse Brain

**Correct image in Additional file 2**:

**Additional file 2. Figure S2:**
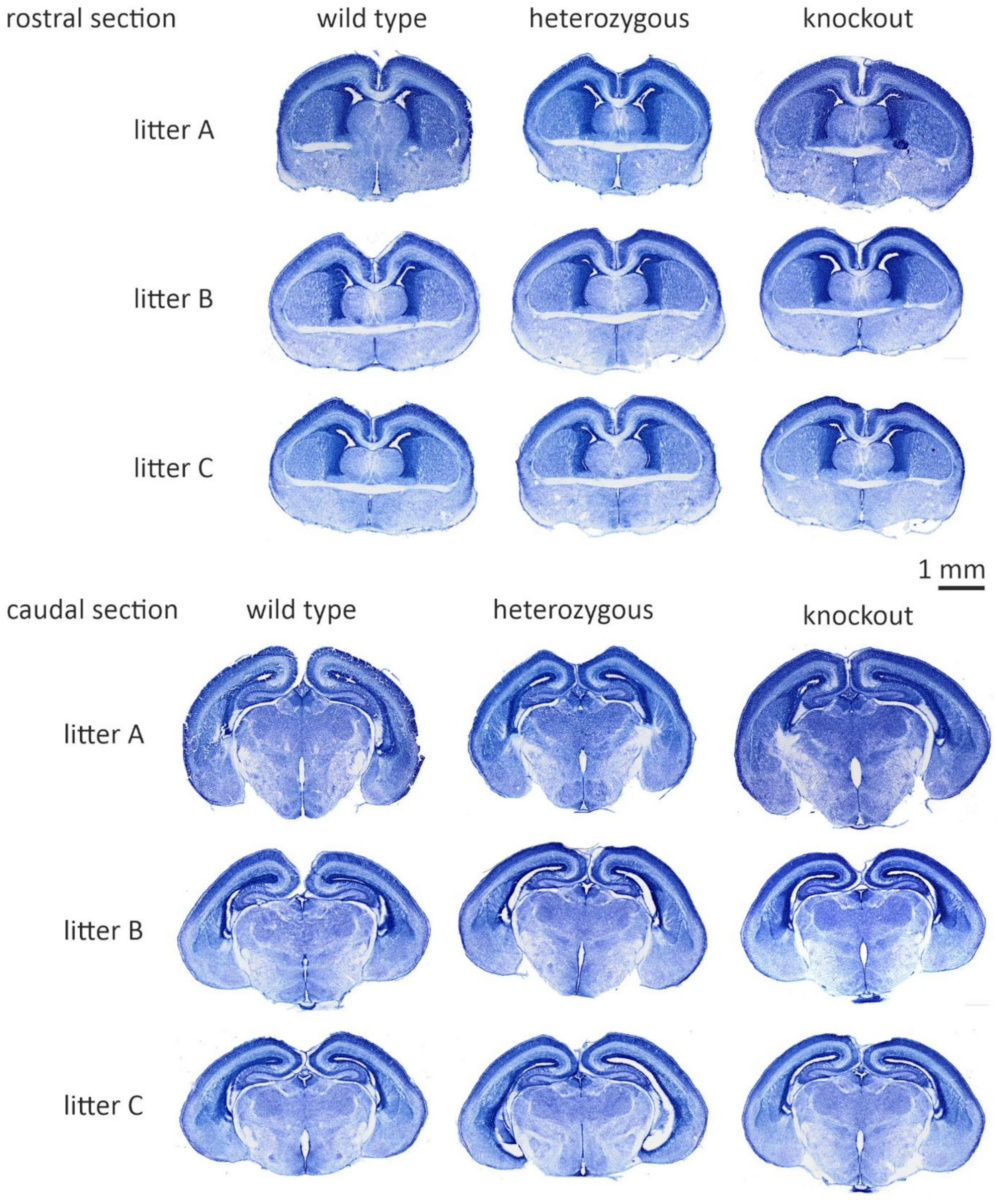
No overt morphological differences between wild-type and *Myt1l* mutant mouse brains. Nissl-stained sections from three animal triplets (*Myt1l*^+/+^, *Myt1l*^+/−^, *Myt1*^−/−^), aged E18.5, from three different litters (*N* = 3/genotype). Examples depict slices corresponding to section 125 (rostral) and 155 (caudal) of the Allen Atlas of the Developing Mouse Brain

