# Supplementary figures and images for: Myt1l haploinsufficiency leads to obesity and multifaceted behavioral alterations in mice
Source: Mol Autism. 2022 May 10;13:19. doi: 10.1186/s13229-022-00497-3 (PMC9087967; doi:10.1186/s13229-022-00497-3)

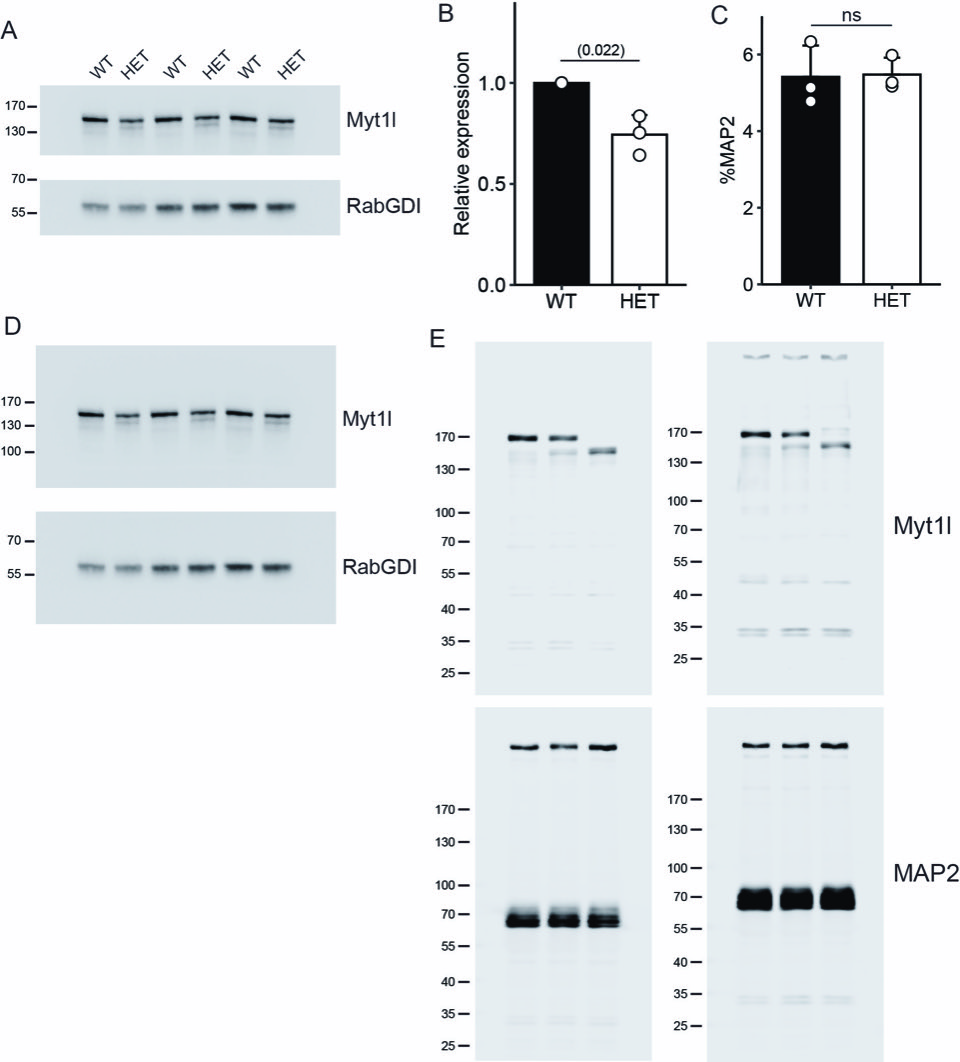

Supplement: Supplementary file 1 — Additional file 1. Figure S1: Molecular characterization of Myt1l expression in mutant mice. (A) Western blot analysis of Myt1l expression in WT or Myt1l HET mouse embryo brains. RabGDI was used as a loading control. (B) Quantification of Myt1l protein expression. The Myt1l bands signal intensity was normalized to that of RabGDI and then to the value of 1.0 for WT (N=3/genotype, one sample t test; P=0.022; error bar, s.d.). (C) qRT-PCR measuring Myt1l transcript levels in brains of WT and HET males (16 month old), normalized to MAP2 (N=3/genotype, t test; ns, not significant, P>0.05; error bars, s.d.). (D) Uncropped Western blot of the blot shown in Suppl. Figure S1A. (E) Uncropped Western blot of the blot shown in Figure 1C. [file 13229_2022_497_MOESM1_ESM.jpeg]

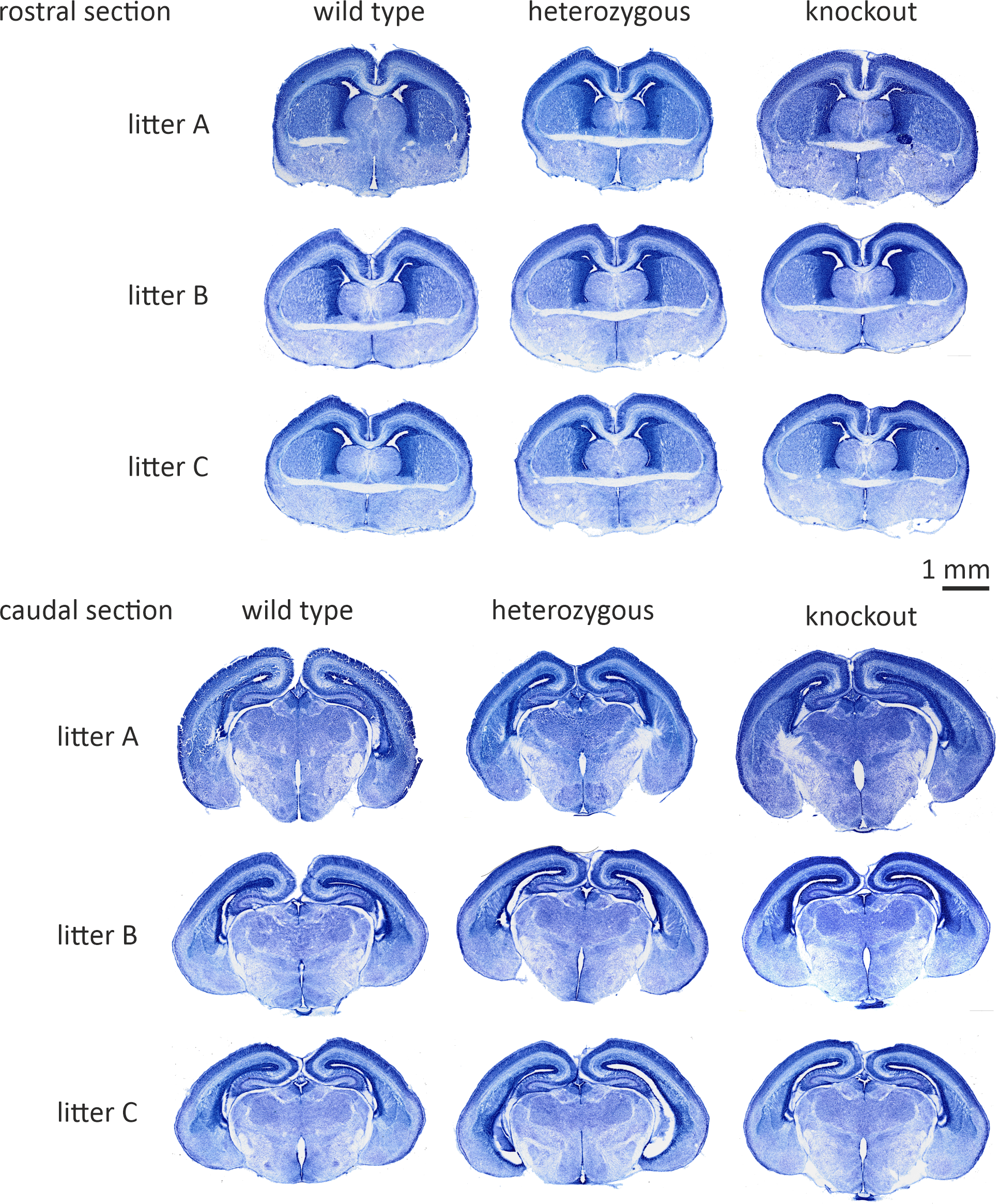

Supplement: Supplementary file 2 — Additional file 2. Figure S2: No overt morphological differences between wild-type and Myt1l mutant mouse brains. Nissl-stained sections from three animal triplets (Myt1l+/+, Myt1l+/-, Myt1-/-), aged E18.5, from three different litters (N=3/genotype). Examples depict slices corresponding to section 125 (rostral) and 155 (caudal) of the Allen Atlas of the Developing Mouse Brain. [file 13229_2022_497_MOESM2_ESM.png]

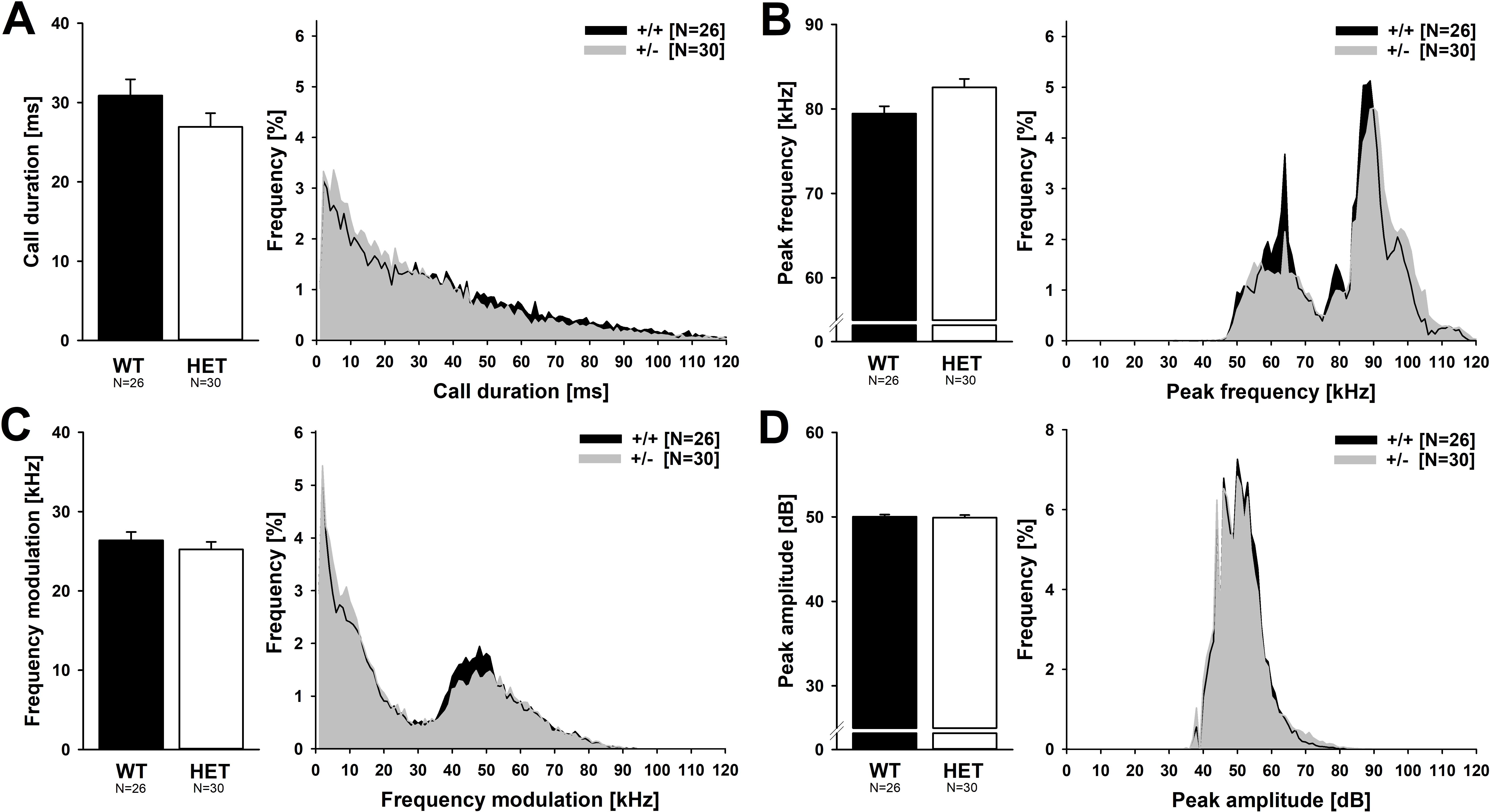

Supplement: Supplementary file 3 — Additional file 3. Figure S3: Effects of Myt1l haploinsufficiency on the emission of isolation-induced ultrasonic vocalizations in the homing test – Acoustic features. (A) Call duration is not altered in Myt1l+/- mouse pups. ANOVA with the between-subject factors genotype (G) and sex (S); all p values > 0.050. (B) Peak frequency is unchanged in Myt1l+/- mouse pups. ANOVA with the between-subject factors genotype (G) and sex (S); all p values > 0.050. (C) Frequency modulation is not affected in Myt1l+/- mouse pups. ANOVA with the between-subject factors genotype (G) and sex (S); all p values > 0.050. (D) Peak amplitude is unchanged in Myt1l+/- mouse pups. ANOVA with the between-subject factors genotype (G) and sex (S); all p values > 0.050. All data are means ± SEM, combined across males and females. [file 13229_2022_497_MOESM3_ESM.jpg]

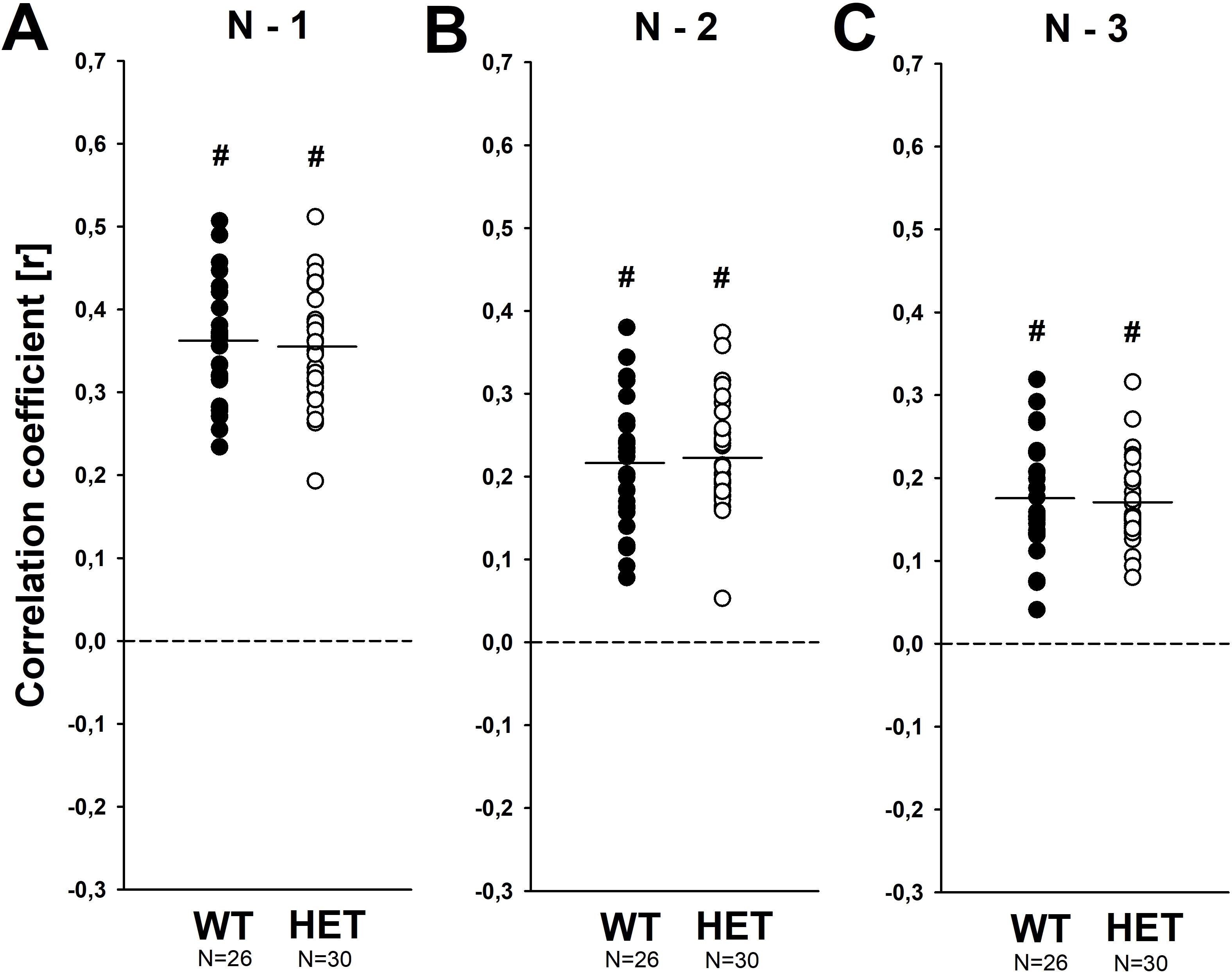

Supplement: Supplementary file 4 — Additional file 4. Figure S4: Effects of Myt1l haploinsufficiency on the emission of isolation-induced ultrasonic vocalizations in the homing test – Temporal Organization. (A-C) Sequential analysis of the durations of subsequent isolation-induced ultrasonic vocalizations indicating a non-random call emission pattern in Myt1l+/- mouse pups. Correlations between the durations of given isolation-induced USV and the durations of the previous ones (N – 1), the durations of the ones two before (N – 2), and the durations of the ones three before (N – 3). Sequential analysis is based on individual isolation-induced ultrasonic vocalizations in Myt1l+/+ littermate controls (~20,000 calls) and Myt1l+/- mouse pups (~30,000 calls), combined across males and females. # = p < 0.05 vs. correlation coefficient r = 0. [file 13229_2022_497_MOESM4_ESM.jpg]

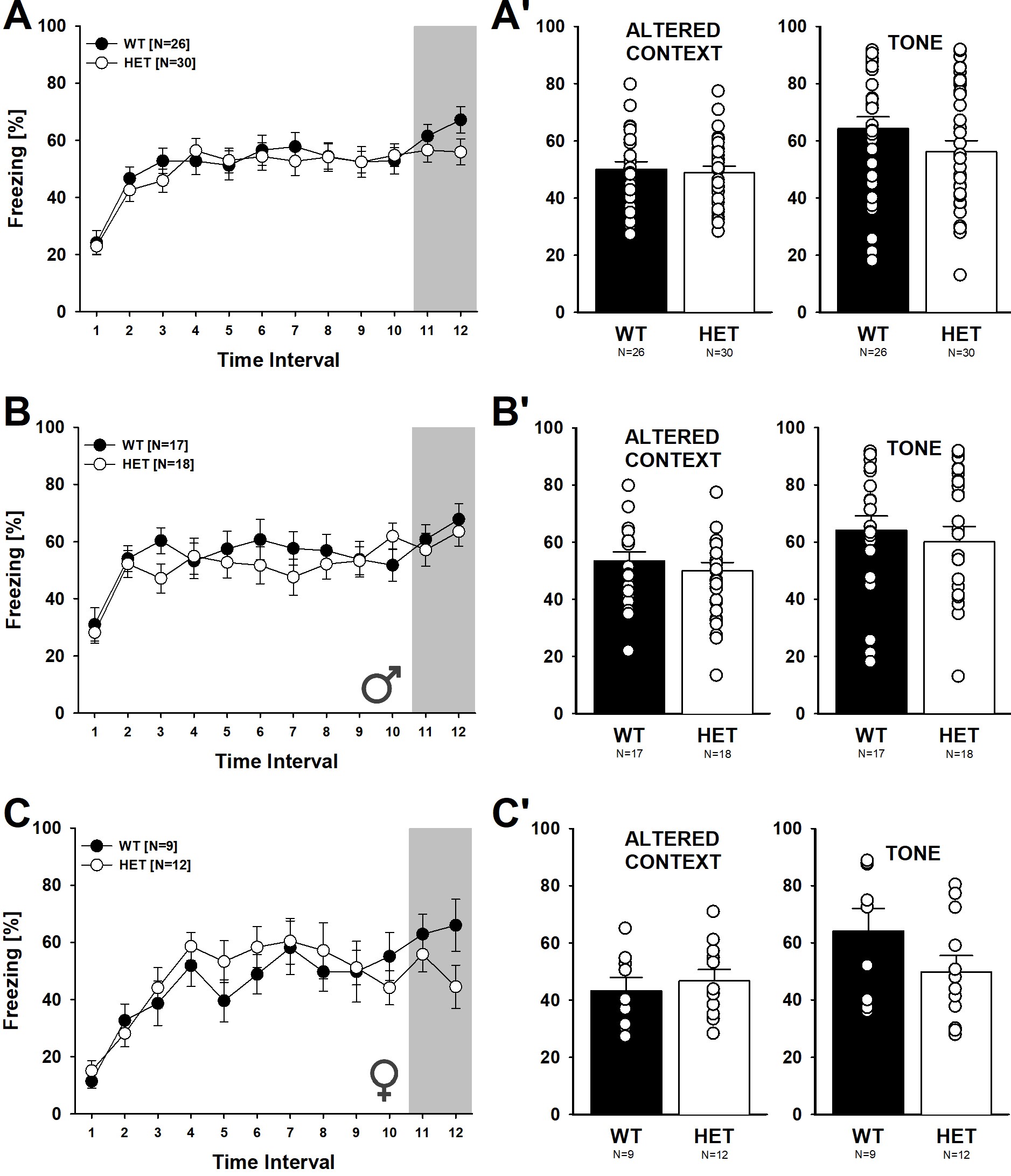

Supplement: Supplementary file 5 — Additional file 5. Figure S5: Effects of Myt1l haploinsufficiency on fear conditioning. (A-C) Fear-related freezing behavior is unchanged in male and female Myt1l+/- mice during cued recall. Gray highlighting indicates tone presentations. Repeated-measures ANOVAs with the between-subject factors genotype (G) and sex (S) and the within-subject factor time (T). T: F11,572=12.667; p<.001; TxS: F11,572=1.996; p=.027; all other p values > 0.050. All data are means ± SEM, combined across males and females if not otherwise indicated. [file 13229_2022_497_MOESM5_ESM.jpg]
